# Extracellular exosome signature links hypertension and Alzheimer’s disease

**DOI:** 10.1007/s11357-026-02097-y

**Published:** 2026-02-04

**Authors:** Joakim A. Bastrup, Jonas Folke, Sanne S. Kaalund, Michel Rasmussen, Susana Aznar, Thomas Jepps

**Affiliations:** 1 Vascular Biology Group, Department of Biomedical Sciences, Faculty of Health and Medical Sciences, University of Copenhagen, Blegdamsvej 3, 2200 Copenhagen N, Denmark; 2 Centre for Neuroscience and Stereology, Department of Neurology, Bispebjerg and Frederiksberg Hospital, Copenhagen University Hospital, 2400 Copenhagen, NV Denmark

**Keywords:** Hypertension, Alzheimer’s disease, Proteomics, Exosomes

## Abstract

Untreated hypertension is a risk factor for late-onset Alzheimer’s disease (AD); however, this association is not well understood. The aim of this study was to reveal protein signatures that bridge the pathophysiological changes in hypertension to AD. Using untargeted proteomics, we analyzed brain samples from aged (30- and 40-week-old) spontaneously hypertensive rats (SHRs, *n* = 12), and age-matched normotensive Wistar Kyoto (WKY, *n* = 8) controls, and human AD patients (Braak stages 4–6) (*n* = 30), cerebral amyloid angiopathy (CAA) (*n* = 5), non-demented controls (Braak stages 0–3) (*n* = 37). Differential expression and pathway analyses in SHRs highlighted the ‘extracellular exosome’ pathway. This pathway also showed significant associations to differentially expressed proteins in AD and CAA patients. Comparison between species identified 24 proteins in SHRs and AD, whose trajectory pattern over the progression of hypertension, aligned with those observed during AD Braak stage progression, compared to respective controls. The proteins were similarly associated with extracellular exosomes. Immunostaining and spatial proteomics support vesicle accumulation and dysregulated exosome protein signatures in the cerebrovasculature of both SHR and AD brains. Additionally, the extracellular exosome pathway-association was not identified in a traditional model of familial AD (5xFAD). Our findings demonstrate cross-species translatability between AD and the SHR and provide novel mechanistic insights into a shared dysregulation of cerebral artery-associated exosomes.

**Graphical Abstract:**

**Figure:**
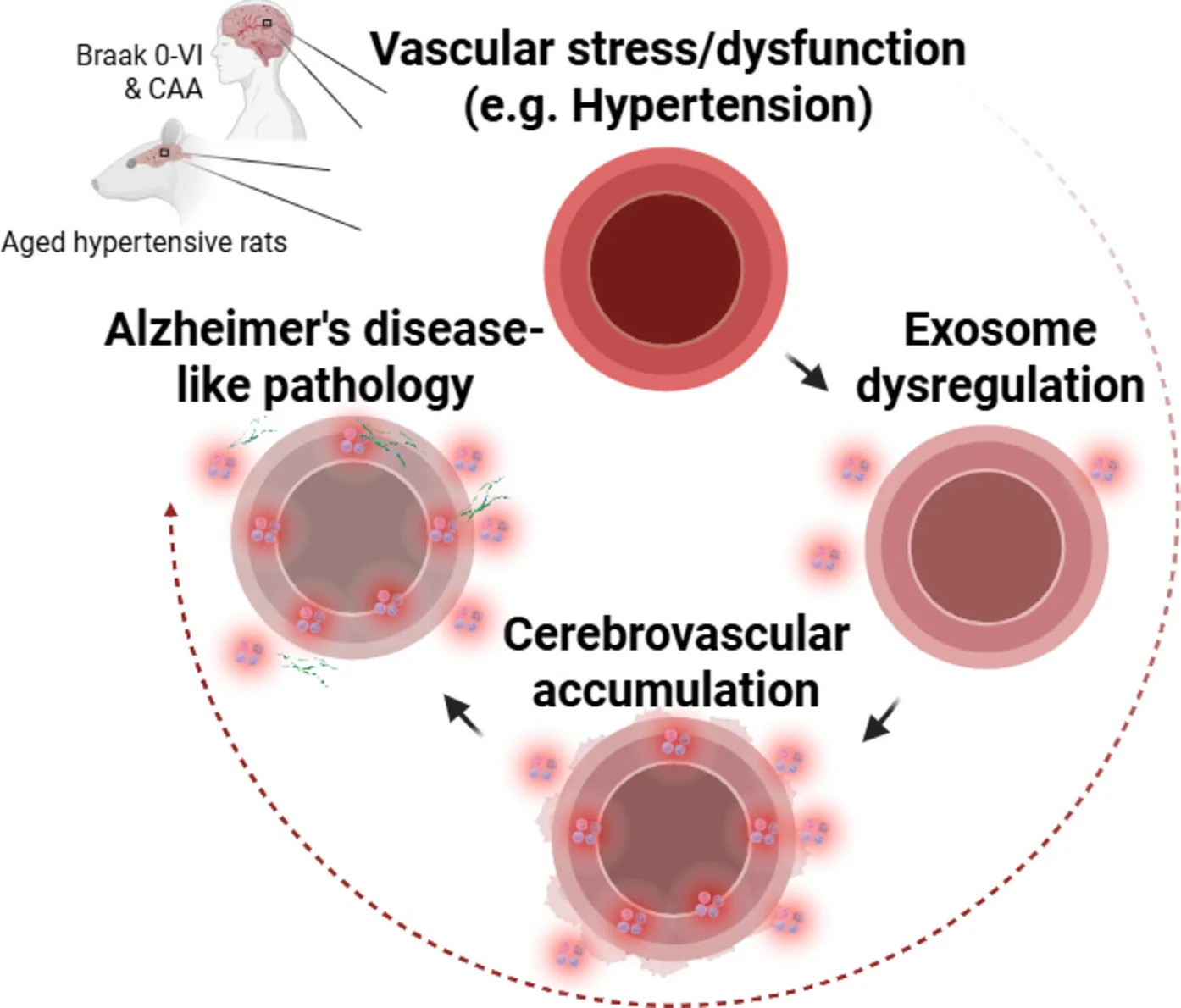

**Supplementary Information:**

The online version contains supplementary material available at https://doi.org/10.1007/s11357-026-02097-y.

## Introduction

Alzheimer’s disease (AD) is the leading cause of cognitive impairment in the elderly. It is characterized by extracellular accumulation of beta-amyloid (Aβ) and intraneuronal tau tangle formation [1]. Cerebrovascular dysfunction is now considered a critical component in prodromal AD pathogenesis, including impaired cerebral blood flow [2–4], increased cerebrovascular resistance [5], and dysfunction of the neurovascular unit and blood–brain barrier [6, 7]. Untreated hypertension causes cerebrovascular dysfunction [8, 9], and several studies report an increased risk of late-life AD in patients with midlife untreated hypertension [10–13]. Furthermore, anti-hypertensive treatment, such as angiotensin II receptor blockers, can reduce the incidence of AD [14]. Despite this association, an in-depth mechanistic understanding that bridges the two diseases is lacking, necessitating further studies to establish the relationship [15].

A key challenge in studying the mechanisms underlying this association has been the lack of representative animal models that capture the development of sporadic, non-familial AD. Currently, research is based primarily on animal models overexpressing autosomal dominant mutations in the genes encoding Aβ; however, this inherited form accounts for only 1–5% of all AD cases [16]. The vast majority of AD cases are sporadic (95–99%) and are associated with a mixture of environmental risk factors, such as obesity, diabetes and hypertension [16, 17].

Previously, the stroke-prone spontaneously hypertensive rat (SHR-SP) was reported to develop an AD-like pathology, including extracellular Aβ plaques [18] and signs of intracellular hyperphosphorylated tau (p-tau) [19]. This phenotype occurs spontaneously and is thought to arise because of chronic arterial hypertension. The SHR-SP model is inbred from the SHR [20] to acquire a stroke-prone phenotype. Since stroke is also associated with AD [21], one limitation of the SHR-SP model is the difficulty to distinguish whether it is the impact of stroke or the exposure to high blood pressure that triggers the AD-like changes. The SHR model without stroke proneness may therefore be a better model in this regard, but a thorough molecular characterization in relation to AD protein hallmarks is lacking. In support of the SHR as a model of AD, the SHR has been reported to exhibit cognitive decline, neuronal loss, angiogenic dysfunction, cerebral hypoperfusion and brain atrophy [22–26].

The aim of this study was to determine whether the SHR model displays protein hallmarks that are mirrored in human AD patients. Herein, we reveal novel proteins and pathway changes in the brains of the SHR that are shared with AD patients, providing critical insight into how cerebrovascular dysfunction may contribute to AD. Notably, the molecular overlap was significantly associated with extracellular exosomes, which was not recapitulated in a conventional animal model of AD. This suggests that the SHR could be considered a novel model that resembles different pathological changes in AD compared to the current available genetic models, thus enabling the development of potential therapeutics targeting different aspects of the disease.

## Methods

A detailed description of Methods can be found in the Supplementary Information.

### Experimental animals

The animal experiments with SHR and Wistar Kyoto (WKY) rats were approved by local Animal Care and Use Committees (institutional approval numbers P23-177 and P24-062). The experiments were conducted in accordance with the directives of the Danish National Committee on Animal Research Ethics and Danish legislation on experimental animals. Rats at 30 or 40 weeks of age were euthanized by decapitation while under anesthesia (SHR, *n* = 12; WKY, *n* = 8). The major cerebral arteries from these rats were also used in a previous study by Bastrup et al. (2025) [27]. In a subgroup of animals (*n* = 2 SHRs and WKYs), the left carotid artery was catheterized for arterial blood pressure measurement. Increased mean blood pressure over a stable 3-min period was confirmed in SHRs compared with WKYs (Mean blood pressure ± standard deviation: SHR = 157.8 ± 12.6, WKY = 99.1 ± 8.3). The brains were carefully excised, rinsed in cold physiological salt solution saturated with carbogen (O_2_ 95%; CO_2_ 5%) at pH 7.4. The brains were divided into two hemispheres, and either snap frozen in liquid nitrogen or fixed in 10% neutral buffered formalin.

### Demographics of human samples

The human brains analyzed in the current study came from two cohorts. Cohort 1 was donated to the Bispebjerg Brain Bank at Bispebjerg-Frederiksberg Hospital (Copenhagen University Hospital, Denmark), and Cohort 2 to the Netherlands Brain Bank for later inclusion at Bispebjerg Brain Bank. Cohort 1 included brain samples from 4 brains with AD pathology and 4 brains with mild primary age-related tau (PART) pathology (Braak stage 1–3, Thal phase 0–1) from non-demented individuals. Cohort 2 included 30 patients diagnosed with AD (ranging from Braak stages 4–6), 5 patients diagnosed with cerebral amyloid angiopathy (CAA), and 37 brain samples from non-demented individuals (ranging from Braak stages 0–3). Demographic analysis of human samples in cohort 2 confirmed similar age, gender, post-mortem interval (PMI), and APOE genotypes (Table 1). The collection and analysis of the samples were conducted in accordance with the World Medical Association Declaration of Helsinki and approved by the regional ethical committee of the Capital Region (Region Hovedstaden, Denmark), j.nr. H-23030276, and the Danish Data Protection Agency (j.nr. p-2023–15234). All donors provided informed and written consent. Brain tissue was stored at − 80 °C until protein extraction.

**Table 1:** Demographics of human cohorts in study. N: number of individuals

Group	*N*	Braak stage	Age (years)	Sex (F/M)	PMI (hours)	APOE3/4	History of hypertension	Mean blood pressure (last recording, mmHg)	Anti-hypertensive medication (*n*)
*Cohort 1*									
Control	4	0–3	91.3 (7.5)	2/2	61 (20.5)	NA	4	131 (18.9)/71.3 (16.3)	Yes (4)
AD	4	5–6	73.8 (5.5)	3/1	55.3 (12.8)	NA	2	133 (24.3)/74.3 (18.9)	Yes (2)
*p*-values	*-*	-	*0.017*^¤^	*0.465*^#^	*0.757*^¤^	*-*	*-*	*0.814*^¤^	*-*
*Cohort 2*									
CB 0	10	0	74.8 (6.2) [64–83]	2/8	6.1 (2.2) [1–8]	7/3	NA	NA	NA
CB 1	10	1	80.8 (5.4) [70–91]	5/5	6.8 (2.3) [4–11]	10/0	NA	NA	NA
CB 2	10	2	83.6 (4.0) [77–89]	5/5	5.6 (1.6) [4–9]	7/3	NA	NA	NA
CB 3	7	3	85.7 (6.3) [74–93]^§^	3/4	6.3 (2.3) [4–11]	4/3	NA	NA	NA
ADB 4	10	4	82.1 (9.2) [58–93]	6/4	4.6 (0.8) [3–6]	6/4	NA	NA	NA
ADB 5	10	5	81.3 (7.9) [63–92]	5/5	4.7 (1.3) [3–7]	5/5	NA	NA	NA
ADB 6	10	6	78.5 (6.3) [65–86]	5/5	4.6 (1.9) [3–9]	4/6	NA	NA	NA
CAA	5	4.8 (0.5) [4, 5]	80.2 (9.3) [67–89]	4/1	4.6 (1.1) [3–6]	2/3	NA	NA	NA
*p*-values	*-*	*-*	0.029*	*0.552*^*#*^	0.036*^,&^	*0.162*^*#*^	*-*	*-*	*-*

*F*, Female; *M*, Male; *PMI*, postmortem interval. Mean; (std); [range]. *: Kruskal–Wallis test and Dunn’s multiple comparison test. ^*&*^: no significance between groups. ^§^: significantly different from CB0. ^#^: chi-squared test. ^¤^: Student's *t*-test.

### Statistical analysis of demographics

Statistical analyses were conducted to assess differences in demographic characteristics across the cohorts and subgroups (Table 1; Supplementary Information Table S1). The variables analyzed included age, sex, postmortem interval (PMI) and APOE genotype.

### Histology

Human and rat samples were fixed in 10% neutral buffered formalin and then embedded in paraffin. Formalin-fixed paraffin-embedded (FFPE) rat and human brain sections (16 µm) were deparaffinized in xylene, hydrated in progressively decreasing concentrations of ethanol and rinsed in running water. Antigen retrieval was performed in citrate-buffer (pH 6) using a microwave. Tissue sections were stained with anti-alpha smooth muscle actin antibody [1A4] (ab7817, produced in Mouse, 1:400, Abcam), anti-CD81 (HPA007234, produced in Rabbit, 1:400, Sigma) and anti-CD31 (AF3628, produced in goat, 1:100 from 1 µg/µl stock 1 × PBS, R&D Systems). Sections were counterstained with secondary antibodies cross-absorbed to Alexa Flour 488, 564, and 647, respectively, (1:400, Invitrogen) for 1 h at RT. Nuclei were stained with Hoechst 33,342 (1:1000 in 1 × PBS, Invitrogen), and sections were mounted in anti-fade mounting medium (Invitrogen, Cat no. S36967).

Fluorescent images were acquired on a LSM900 confocal imaging setup (Zeiss) with a 20 ×/0.8 Plan-Apochromat Air objective lens (Zeiss). Statistical analyses were made in GraphPad Prism (v10) using unpaired Student’s *t*-test.

### Sample preparation for proteomics

Tissues (approximately 0.3 cm × 0.3 cm × 0.3 cm) were homogenized in 300 μl of ice-cold lysis buffer [28]. The homogenates were centrifuged at 11,000 g for 10 min and protein quantification of the tissue extracts was determined by bicinchoninic acid (BCA) assay (Thermo Fisher Scientific, cat no. 23227). Tissue extracts (50 μg protein) were heated to 70 °C for 10 min. The samples were prepared using filter-aided sample preparation (FASP) [29] with flat spin filters (Microcon-10 kDa, Cat no. MRCPRT010). The samples were reduced and alkylated in digestion buffer containing 1:50 (v:v) tris(2-carboxyethyl)phosphine (TCEP; 0.5 M, Sigma,) and 1:10 (v:v) 2-chloroacetamide (0.5 M, Sigma,) for 30 min at 37 °C. Proteolytic digestion was performed overnight at 37 °C by addition of 0.5 μg Trypsin/LysC mix (Promega, cat no. V5072) (at enzyme to protein ratio 1:100). The resulting peptides were desalted and stored at 80 °C until further analysis [26].

### Sample preparation for laser microdissection

Polyethylene naphthalate (PEN) membrane slides (Applied Biosystems™, LCM0522) were coated with Vectabond (Vector Laboratories, Cat. No. SP-1800–7) following the manufacturer’s instructions. FFPE tissue sections were mounted onto pre-treated slides and dried at RT. Slides were deparaffinized, rehydrated and subjected to antigen retrieval as described in the *Histology* section. Sections were stained with anti-alpha smooth muscle actin antibody [1A4] (ab7817, produced in Mouse, 1:400, Abcam) and counterstained with anti-mouse HRP-conjugated EnVision + detection reagent (DAKO, Cat. No. K4001) for 30 min at RT. Signal development was performed using the 3,3′-diaminobenzidine (DAB) Bright system (ImmunoLogic, Cat. No. VWEKBS04-500) for 8 min. Slides were then rinsed in distilled water, stained with Mayer’s hematoxylin for 30 s, wash under running tap water for 7 min and rinsed in deionized water for 3 min. The membrane was punctured by creating small holes and dried for 45 min at 60 °C followed by storage at RT until laser dissection.

Regions of interest (artery, capillary or adjacent parenchyma) were cut using the LMD7 (Leica) laser microdissection system with the following settings: Power = 30, Aperture = 15, Speed = 20, Specimen balance = 15, Head current = 100%, Puls frequency = 120. The microscope was operated with the LMD v8.2 software, with ThreeSlides sample holder and the standard PCR tube collector (4 tubes). A total tissue area of approximately 200,000 µm^2^ were collected per region into a 0.2 ml thin walled and flat capped PCR tube (Axygen, PCR-02-C). The dissected elements were confirmed by visual inspection under microscope. Tubes were centrifuged at 5000 g for 10 min, then 25 ul of digestion buffer was added to the cap and tubes were centrifuged again. Tubes were heated for 1 h at 95 °C in a heat block, cooled to RT and centrifuged for 30 s at 5000 g. Samples were reduced and alkylated by adding 0.5 ul TCEP (0.5 M, Sigma) and 2.5 ul CAA (0.5 M, Sigma) followed by 20 min incubation at 37 °C. Subsequently, 20 ng of Trypsin/LysC mix (Promega, cat no. V5072) was added and proteins were digested overnight at 37 °C. Samples were desalted as described in the “Sample preparation for proteomics” section and subsequently reconstituted in 7 ul reconstitution buffer.

### Data acquisition by mass spectrometry

Peptide mixtures were analyzed on Bruker timsTOF mass spectrometer using positive ion mode, with a Captivespray ion source on-line connected to a Dionex Ultimate 3000RSnano chromatography system (Thermo Fisher). Peptides were separated on an Aurora column with captive spray insert (C18 1.6 uM particles, 15 cm, 75 μm inner diameter, IonOptics; For LMD samples, C18, 1.7 uM particles, 8 cm, 150 μm inner diameter, IonOptics) at 60 °C. A solvent gradient of buffer A (0.1% formic acid) and buffer B (99.9% acetonitrile/0.1% formic acid) was used over 22 min for human samples and 28 min for rat samples, at a flow rate of 400 nL/min and 600 nL/min for human and rat samples, respectively. The mass spectrometer was operated in DIA PASEF mode with 1.1 s cycle time and TIMS ramp time of 100.0 ms. MS scan range was set to 100–1700 m/z. For LMD samples, a 15-min gradient was used and high sensitivity was enabled.

### Protein identification by mass spectrometry

Raw DIA files were analyzed with DIA-NN software (v.1.8.1) separately (https://github.com/vdemichev/diann) [30]. An in silico DIA-NN predicted spectral library was generated using UniProt FASTA databases for *Rattus norvegicus*; (UP000002494_10116.fasta and UP000002494_10116_additional.fasta, downloaded August 2020), and for *Homo sapiens*; (UP000005640_9606 and UP000005640_9606_additional, downloaded April 2022).

### Bioinformatics

Protein quantities obtained from DIA-NN were analyzed using Perseus (v1.6.14.0) [31]. A Two-sided Student’s *t*-test was conducted. Enrichment analysis of differentially expressed proteins identified from the SHR versus WKY comparisons and overlapping candidate proteins was performed with STRING app (v2.0.1) [32] in Cytoscape (v3.9.1) [33]. The GO: Biological process and GO: Cellular Component (species = homo sapiens) categories were used. Confidence score cutoff of 0.3 was used for the PPI network generation.

### Western blot

Isolated protein from rat brains was dissolved in SDS-sample buffer containing 0.1 M DTT and heat-treated for 10 min at 70 °C. Proteins were separated using gel electrophoresis on 4–12% Bis–Tris SDS-PAGE gels (Invitrogen) and transferred onto polyvinylidene difluoride (PVDF) nitrocellulose membranes (Immobilon-FL, Millipore). After blocking, they were incubated with primary antibodies, anti-alpha smooth muscle cell actin (ab32575, 1:2500, Abcam) and anti-CD81 (HPA007234, 1:500, Sigma). The membranes were incubated with conjugated secondary antibodies (α-Rabbit 680 or 800, Li-Cor Biosciences; 1:10,000, respectively) for 1 h at RT. The Odyssey Infrared Imaging System (Li-Cor Biosciences) was used for protein visualization and supplier’s software (Image Studio Lite, v5.2.5) was used for analysis.

### Data availability

The mass spectrometry proteomics data have been deposited to the ProteomeXchange Consortium via the PRIDE [34] partner repository with the dataset identifiers PXD057298 and PXD066265.

## Results

### Chronic hypertension in aged rats drives amyloidogenic, tau and proteomic alterations

**Fig. 1:**
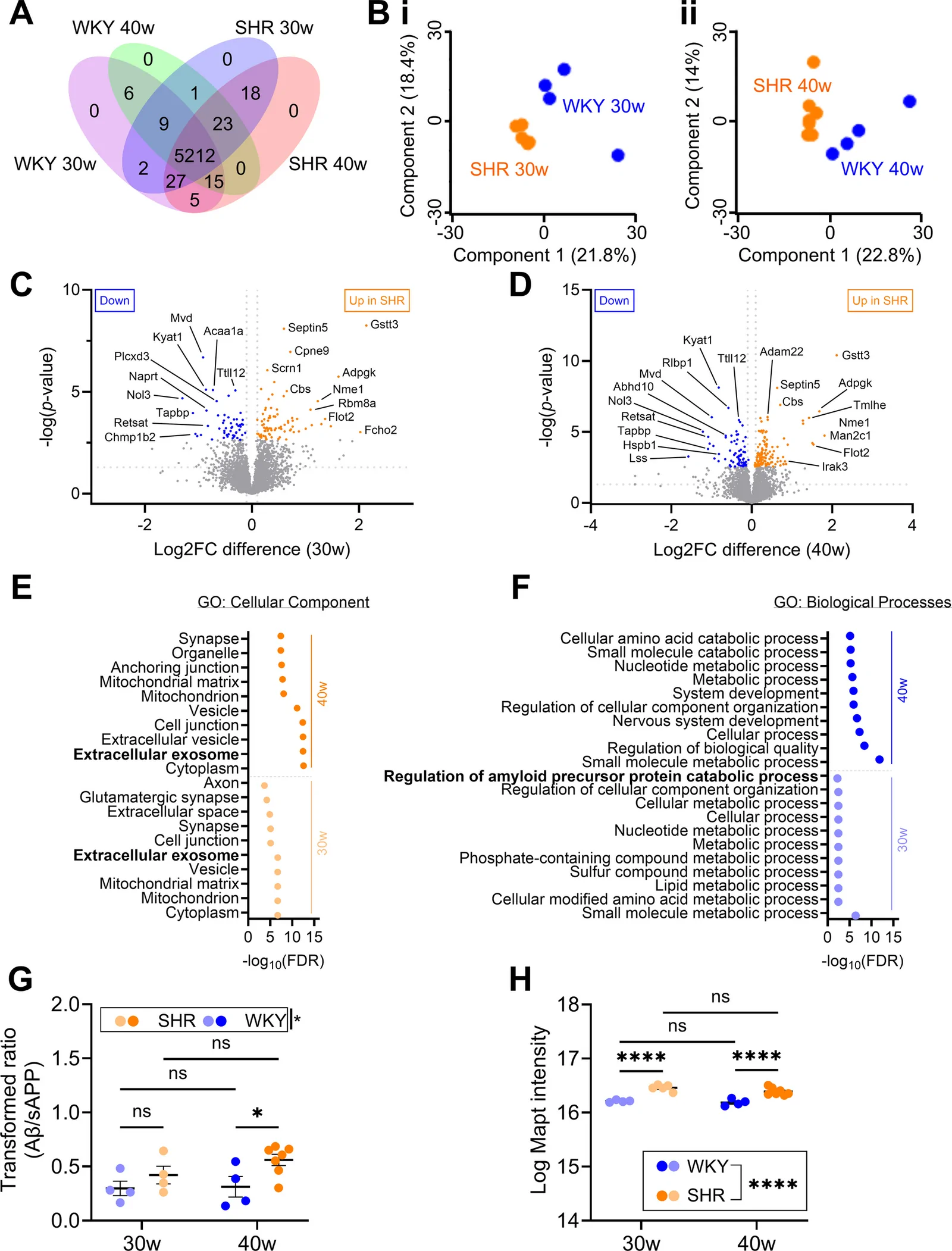
Identification of differentially expressed proteins in the frontal cortex of aged hypertensive rats. (**A**) Venn diagram representation of shared proteins identified by liquid chromatography–tandem MS (LC–MS/MS) using data-independent acquisition (DIA) across the frontal cortex from 30- and 40-week-old spontaneously hypertensive rats (SHR; *n* = 5 and 7) and Wistar Kyoto control (WKY; *n* = 4). (**B**) Principal component analysis (PCA) plot of log2-transformed intensities associated with the 30- (i) and 40-week-old (ii) samples. Components 1 and 2 are presented, SHR = orange, WKY = blue. (**C**) and (**D**) volcano plot comparing protein abundance in frontal cortex samples from 30- (**C**) and 40-week-old (**D**) SHR and WKY controls. Log2 fold change differences and non-adjusted *p*-values are presented. Red = significantly upregulated and blue = significantly downregulated in SHR compared to WKY (adjusted *p*-value < 0.05 cut-off using pairwise mutation-based false-discovery rate (FDR)). (**E) and (F**) Gene overrepresentation analysis of differentially expressed proteins identified from the SHR versus WKY comparisons (GO: Cellular Component and GO: Biological Process categories, respectively). Log10 transformed FDR values (Benjamini–Hochberg procedure) are presented together with annotated ontology term. (**G**) Amyloid beta peptide to soluble Amyloid precursor protein (App) ratio comparison in SHRs (orange) and WKY control (blue). One 30-week-old SHR sample was excluded from the analysis because the specific amyloid beta core peptide with + 2 charge state was not detected. (**H**) Log2 transformed intensities of Total tau (Mapt) detected in DIA analysis in SHR and WKY rat samples. Data was analyzed using Two-way ANOVA and Fisher’s LSD post hoc test, * *p* < 0.05, **** *p* < 0.0001

We investigated the protein composition of rat frontal cortex homogenates by label-free data-independent acquisition (DIA)-MS quantification. Our analysis identified > 5000 unique proteins that were shared between the four groups (Fig. 1A). Using principal component analysis (PCA), we observed clusters corresponding to the phenotypes (SHR and WKY) along component 1, accounting for 21.8% and 22.8% of the variation at 30- and 40-week old, respectively (Fig. 1B). At 30 and 40 weeks of age, SHRs exhibited progressively divergent proteomic profiles compared with age-matched normotensive WKY rats (Fig. 1C, D), with 132 and 199 differentially expressed proteins (DEPs), respectively (Adjusted *p*-value calculated using pairwise mutation-based false discovery rate (FDR) test < 0.05; Supplementary Table S2). GO Cellular Component enrichment analysis revealed a strong and consistent association with the ‘extracellular exosome’ term at both time points (Fig. 1E). Moreover, at 30 weeks, the DEPs were significantly enriched in the GO Biological Process term ‘regulation of amyloid precursor protein (APP) catabolic process’ (Fig. 1F).

The presence of AD protein hallmarks was supported by both quantifying the intensity ratio between the cleaved Aβ peptide and an upstream peptide from the full-length soluble APP (sAPP), as well as by measuring total tau levels. This approach showed significantly increased ratios of Aβ to sAPP, as well as total tau (Mapt) levels, in SHR brains compared with WKY brains (Fig. 1G, H), supporting an AD-like protein hallmark signature.

### Proteomic profiling of human superior and medial frontal gyrus reveals accumulative extracellular exosome changes in AD patients consistent with SHR findings

We investigated the protein composition of human post-mortem brain tissue (*n* = 72) from the superior and medial frontal gyrus, representing the pathological progression from Braak stage 0–6, as well as cases with cerebral amyloid angiopathy (CAA).

**Fig. 2:**
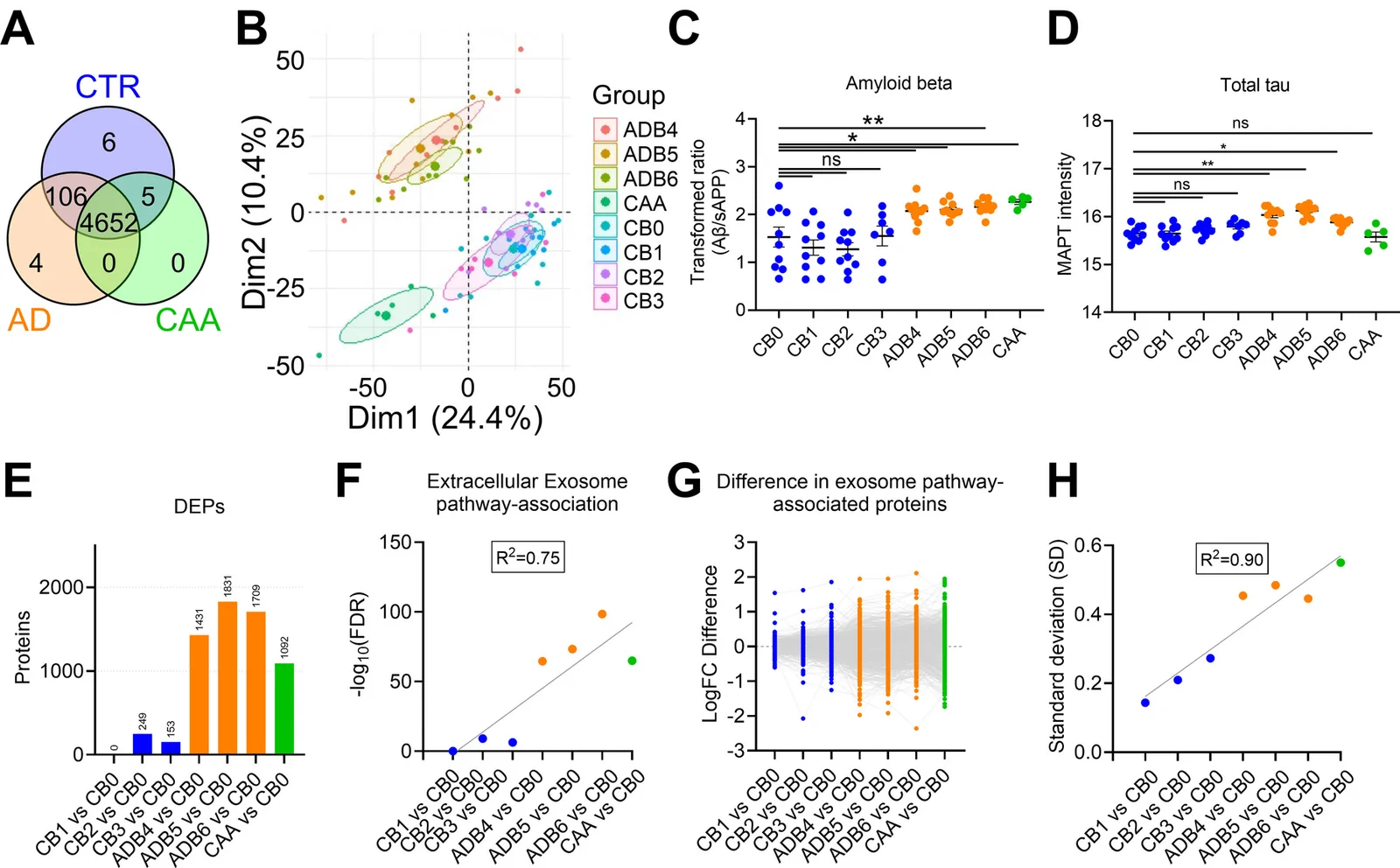
Identification of differentially expressed proteins in superior and medial frontal gyrus of Braak phase 0.6. (**A**) Venn diagram representation of shared proteins identified by liquid chromatography–tandem MS (LC–MS/MS) using data-independent acquisition (DIA) in non-demented control (CTR), Alzheimer’s disease (AD), and cerebral amyloid angiopathy (CAA) samples. (**B**) Principal component analysis (PCA) plot of log2-transformed intensities associated with the samples. Dimensions 1 and 2 are presented. (**C**) Amyloid beta peptide to soluble Amyloid precursor protein (APP) ratio comparison in Braak stages 1–6 and CAA compared to braak 0 (non-demented controls = blue, AD = orange, CAA = green). (**D**) Log2 transformed intensities of Total tau (MAPT) detected in DIA analysis in samples. Data were analyzed using a One-way ANOVA followed by Dunnett’s multiple comparisons test; * *p* < 0.05, ** *p* < 0.01, ns = non-significant *p* > 0.05. **E**) Bar chart depicting number of differentially expressed proteins (DEPs) identified based on *t* test analysis with pairwise mutation-based false-discovery rate (FDR) correction (adjusted *p* value cut-off < 0.05). (**F**) scatter plot representation depicting results from gene overrepresentation analysis of DEPs identified using the GO: Cellular Component category. Log10 transformed false-discovery rate (FDR) values (Benjamini–Hochberg procedure) are presented together with the annotated ontology term ‘Extracellular Exosome’. (**G**) Log2 fold change (logFC) difference of all exosome-associated proteins identified across the different Braak stage and CAA groups. (**H**) standard deviation (SD) based on logFC of all exosome-associated proteins within each group. Simple linear regression lines are depicted in grey

Our proteomic analysis identified more than 4600 unique proteins that were shared between the non-demented control (CTR), AD and CAA samples (Fig. 2A). Using PCA, we observed clusters corresponding to AD (Braak 4–6) and non-demented control (Braak stage 0–3) along component 2 (Dim2), accounting for 10.4% variation in the dataset (Fig. 2B). The Aβ to sAPP ratio levels were significantly elevated in human AD and CAA samples (Fig. 2C). Total tau levels were only significantly increased in AD patients and not CAA or non-demented controls (Fig. 2D). Subsequent deconvolution analysis supported that the increased tau levels did not reflect neuronal or glial proportion changes (Supplementary Information Fig. S1).

The proteomic difference seen in the PCA was supported by DEP counts increasing markedly at Braak stages 4–6 compared to non-demented controls (Braak 0 vs 1 = 0; Braak 0 vs 2 = 249; Braak 0 vs 3 = 153; Braak 0 vs 4 = 1431; Braak 0 vs 5 = 1831; Braak 0 vs 6 = 1709; Braak 0 vs CAA = 1092; Adjusted *p*-values calculated using pairwise mutation-based false discovery rate (FDR) test < 0.05; Fig. 2E; Supplementary Table S2). Using the GO Cellular Component category, a strong association with ‘extracellular exosome’ was observed in AD and CAA samples, which correlated positively with stage severity (R^2^ = 0.75; Fig. 2F). Notably, this observation was supported by increased fold-change differences of the exosome-associated proteins (Fig. 2G) as well as increased standard deviation across the Braak stage progression (R^2^ = 0.90; Fig. 2H), supporting a progressive dysregulation of the exosome signature during pseudo-Braak stage development.

**Fig. 3:**
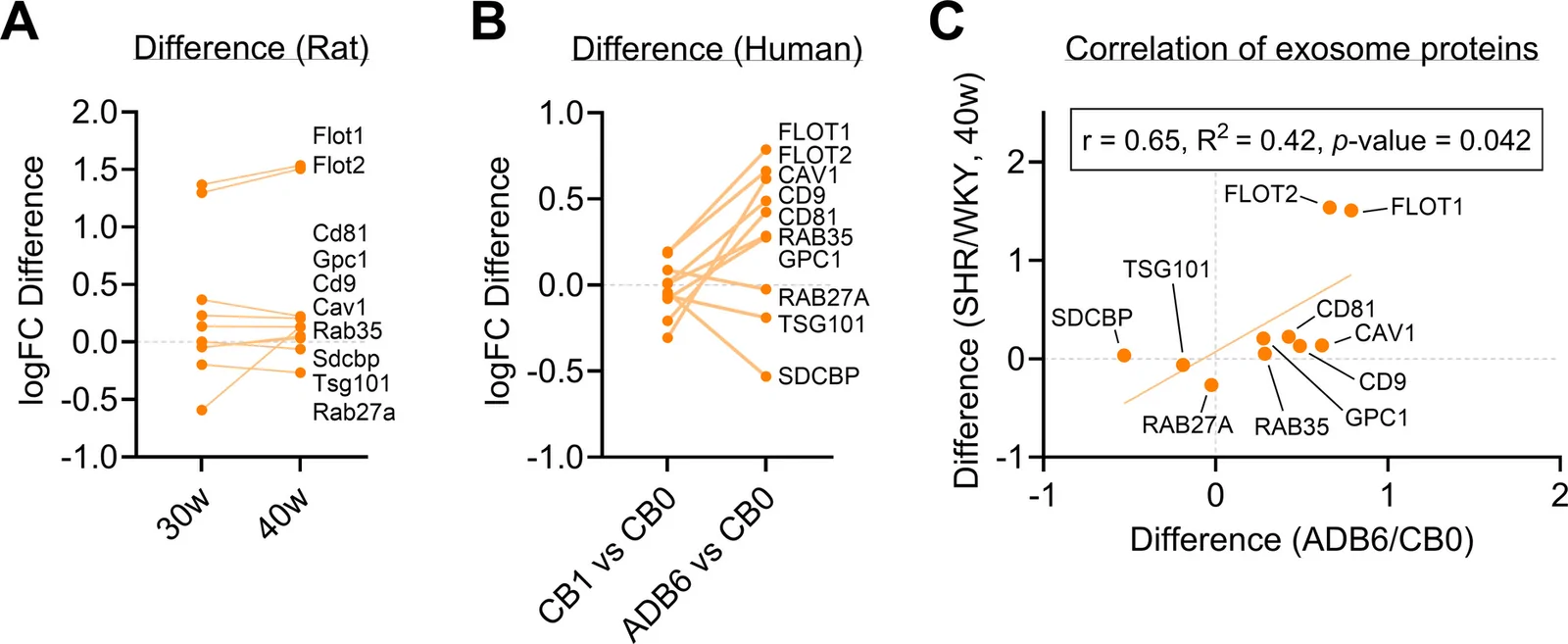
Exosome marker changes in Alzheimer’s disease (AD) brain samples correlates with spontaneously hypertensive rat (SHR) findings. (**A**) Trajectory of fold change differences for selected exosome marker proteins in SHRs compared to Wistar Kyoto (WKY) controls at 30 and 40 weeks of age. (**B**) Similar trajectory plots showing fold-change differences between Braak stage 1 versus Braak 0 and AD Braak 6 versus Braak 0 human samples. (**C**) Correlation between fold change differences in exosome markers proteins from 40-week-old SHR and those from AD Braak stage 6

To further investigate the observed exosome pathway association, we focused our analysis on the fold change differences of selected exosome markers in the datasets from both human and rat (Fig. 3A, B). This showed that while the difference varies among the exosome markers, the general trend was an increase compared to control, which was replicated in both SHR and AD brains. This association was supported by correlation analysis showing significant correlation between the AD Braak 6 stage and SHRs at 40 weeks (Fig. 3C).

### Proteomic comparison identifies 24 candidate proteins in hypertensive rat model, whose trajectory pattern is shared with AD Braak staging

**Fig. 4:**
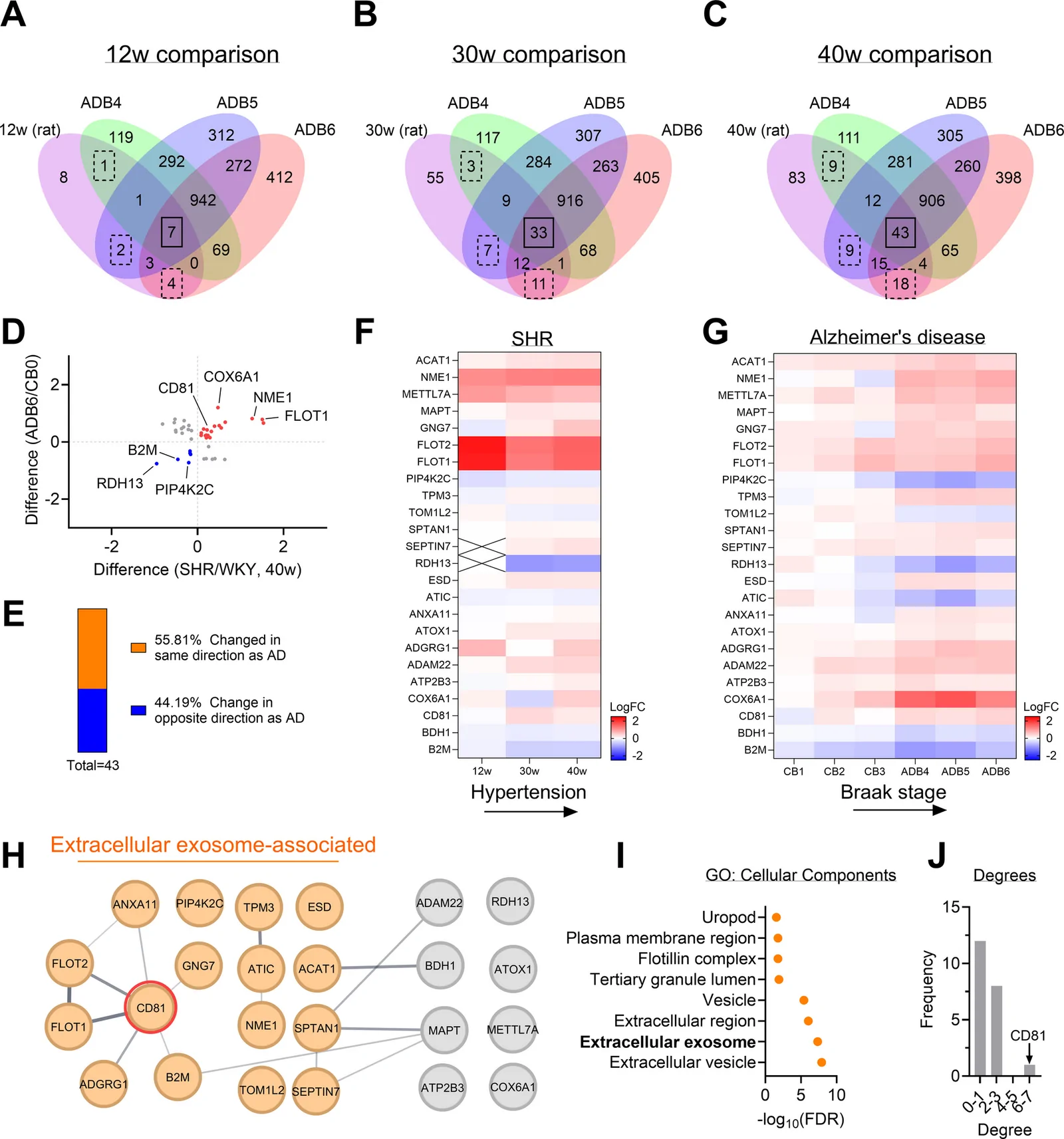
Identification of 24 candidate proteins. (**A**–**C**) Venn-diagram comparing lists of differentially expressed proteins from human and rat comparisons. (**D**) Scatter plot depicting fold change difference from previous comparisons (SHR versus WKY on x axis, and Alzheimer’s disease Braak 6 versus non-demented control Braak 0 on y axis). Red = shared upregulated proteins, and blue = shared downregulated proteins. (**E**) Representation of the number of overlapping proteins that regulated in same direction (orange) or opposite direction (blue). (**F**) and (**G**) Heatmaps depicting log fold change difference for each candidate protein in rat (**F**) and human (**G**), respectively. Bar = log2 fold change difference (± 2.5). (**H**) Protein–protein interaction (PPI) network of candidate proteins. Proteins associated with the ‘extracellular exosome’ pathway term are highlighted in orange. (**I**) Gene overrepresentation analysis of shared candidate proteins using the GO: Cellular Component category. Log10 transformed false-discovery rate (FDR) values (using Benjamini–Hochberg procedure) are presented together with annotated ontology term. (**J**) Bar chart depicting number of proteins in PPI network with 0–1, 2–3, 4–5, 6–7 degrees

To identify conserved AD-related mechanisms, we compared DEPs across SHRs and human AD brains. Initially, we included our previously published data containing information about the protein regulation in the frontal cortex at the early-onset of hypertension (SHR at 12 weeks of age) [26]. From this data set, we identified 7 proteins shared with Braak stages 4–6 (Fig. 4A). This number increased to 33 and 43 shared proteins when comparing DEPs at 30 and 40 weeks of age, respectively, suggesting a progressive blood pressure-dependent effect (Fig. 4A–C).

Of the 43 shared proteins at the 40-week time-point, we identified 24 candidate proteins that were regulated in the same direction in both rats and humans (Fig. 4D, E). All candidate proteins, except Cox6A1, were present in an AD database [35] (Supplementary Information Table S3). However, Cox6A1 has been associated with AD previously [36].

Using a heatmap based on LogFC differences, we tracked the trajectory of protein change in both the SHR model (from early-onset to chronic hypertension) and human AD development (from non-demented Braak 0 to AD Braak 6) (Fig. 4F, G). For most proteins, there was a progressive temporal change in rats and with Braak staging in humans. One example is MAPT (total tau), which increased over time according to Braak staging progression and hypertension in the SHRs (Fig. 4F, G).

Protein–protein interaction (PPI) network and enrichment analysis of these shared DEPs highlighted a significant association with ‘extracellular exosome’, in which 16 out of 24 proteins (66.6%) were associated (Fig. 4H, I). Notably, when analyzing the number of direct interactions (degrees) in the PPI network, one protein, CD81, had 6 interactions and was thus considered an important hub in the network that could play a critical role in regulating the exosome pathway (Fig. 4J).

### Artery-associated exosome dysregulation bridge hypertension and AD

**Fig. 5:**
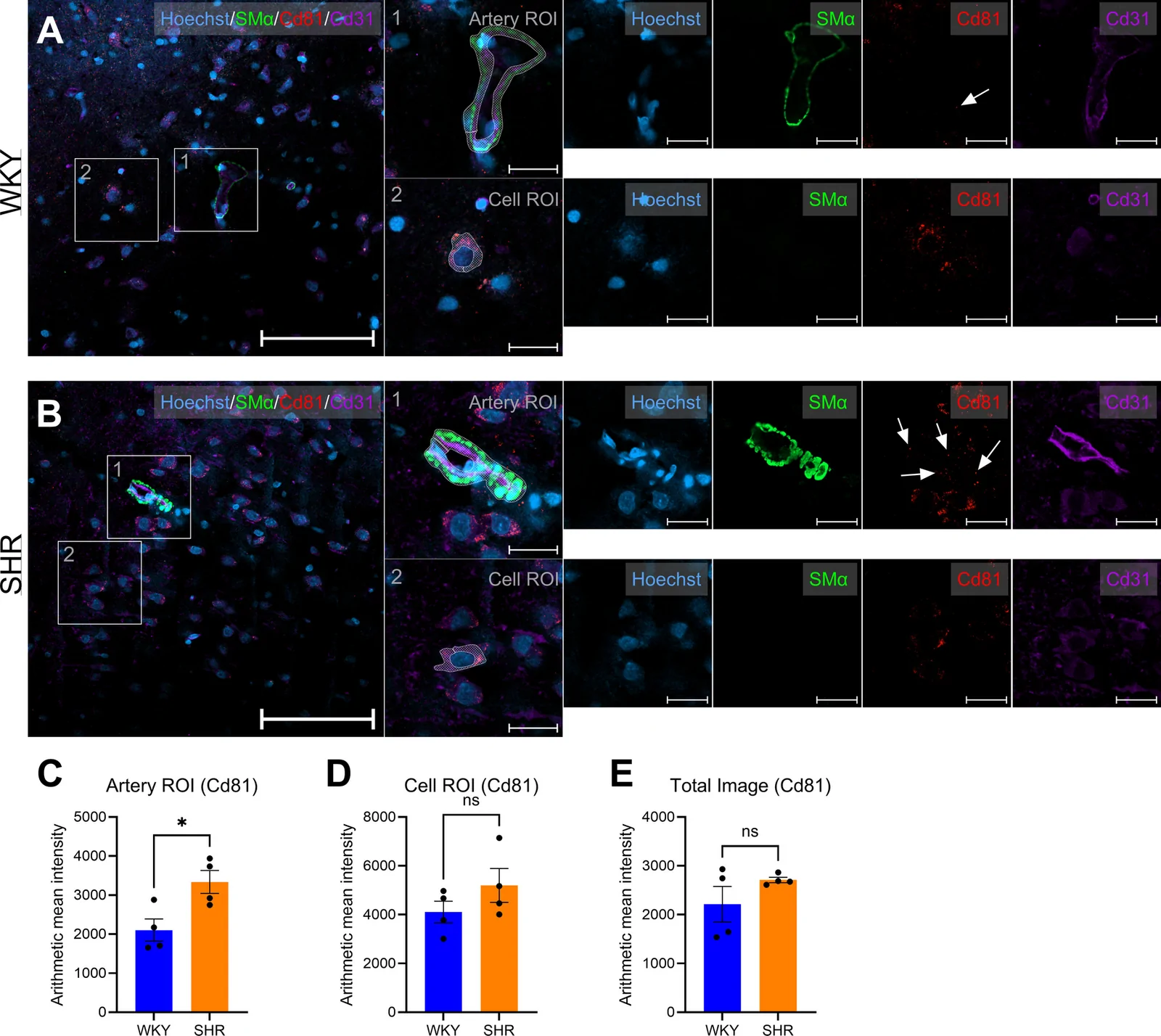
Cd81 expression is increased in arterial wall of hypertensive rats. Immunohistochemistry analysis of α-SMA (green), CD81 (red), CD31 (magenta) in frontal cortex of 40-week-old Wistar Kyoto (WKY) control (**A**) and spontaneously hypertensive rats (SHR) (**B**). (**C**–**E**) Cd81 fluorescent signal in artery, adjacent cell or total image, respectively (n = 4 biological replicates with 1–2 arteries per replicate; Student t test, * *p* value < 0.05). Images were obtained with a 20 × objective lens. Scale bar = 100 µm large image, and 20 µm in small region-of-interest (ROI) images. Arrows mark sites of artery-associated Cd81 location. SEM is included in column graphs (WKY = blue, SHR = orange)

**Fig. 6:**
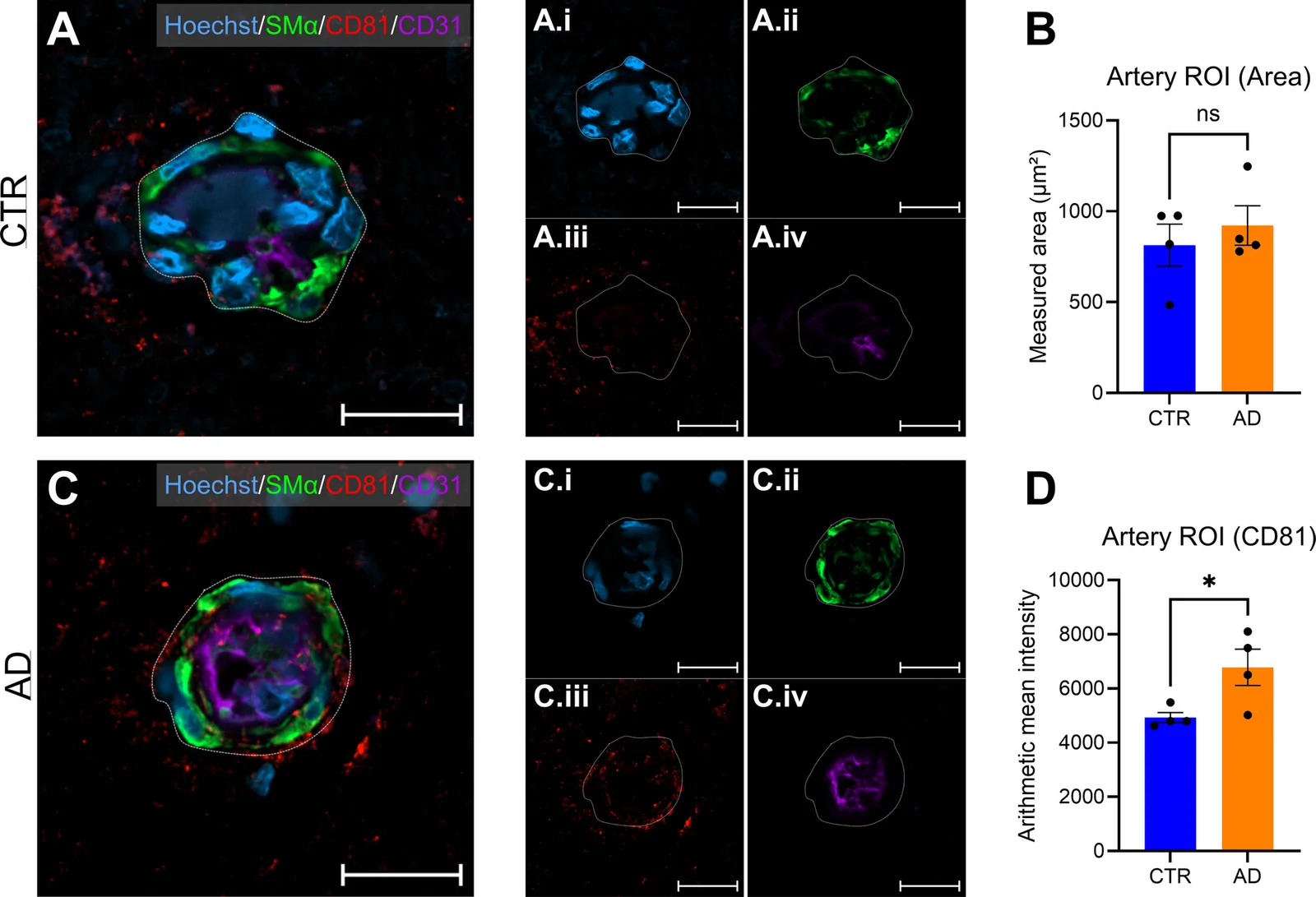
CD81 expression in cerebral arteries in human Alzheimer’s disease (AD) patients and controls. Immunohistochemistry analysis of α-SMA (green), CD81 (red), CD31 (magenta) in occipital cortex from AD-hallmark (p-tau and Aβ plaques) positive patients (**C**) and hallmark-negative controls (**A**). (**B**) and (**D**) CD81 analysis performed similarly to (Fig. 5) by quantifying the fluorescent mean intensity within a region of interest (white dotted lines); n = 4 biological replicates with 1–3 arteries per replicate; Student *t* test, * *p* value < 0.05. Images were obtained with a 20 × objective lens and cropped to illustrate single cerebral arteries. Scale bar = 20 µm

Focusing on the exosome pathway, we validated upregulation of the extracellular vesicle-associated protein CD81 in both SHRs and human AD patients. In SHRs, Western blot and immunostaining revealed elevated CD81 expression localized to vascular smooth muscle and endothelial cells (Fig. 5A–E; Supplementary Information Fig. S2), with no change in the parenchyma. In an additional cohort of AD brains and brains from non-demented controls (*n* = 4 in each group), this vascular-specific expression was mirrored, with CD81 increased in cerebral arteries of AD patients compared to controls (Fig. 6A–D). No differences were observed in the immunofluorescent intensities from CD31 (endothelial cell marker), smooth muscle alpha-actin (SMα; vascular smooth muscle cell marker) and Hoechst, or in the measured region of interest area in both stainings, respectively (Supplementary Information Fig. S3). An alternative lookup table (LUT) representation of the artery-associated CD81 localization is provided in (Supplementary Information Fig. S4).

**Fig. 7:**
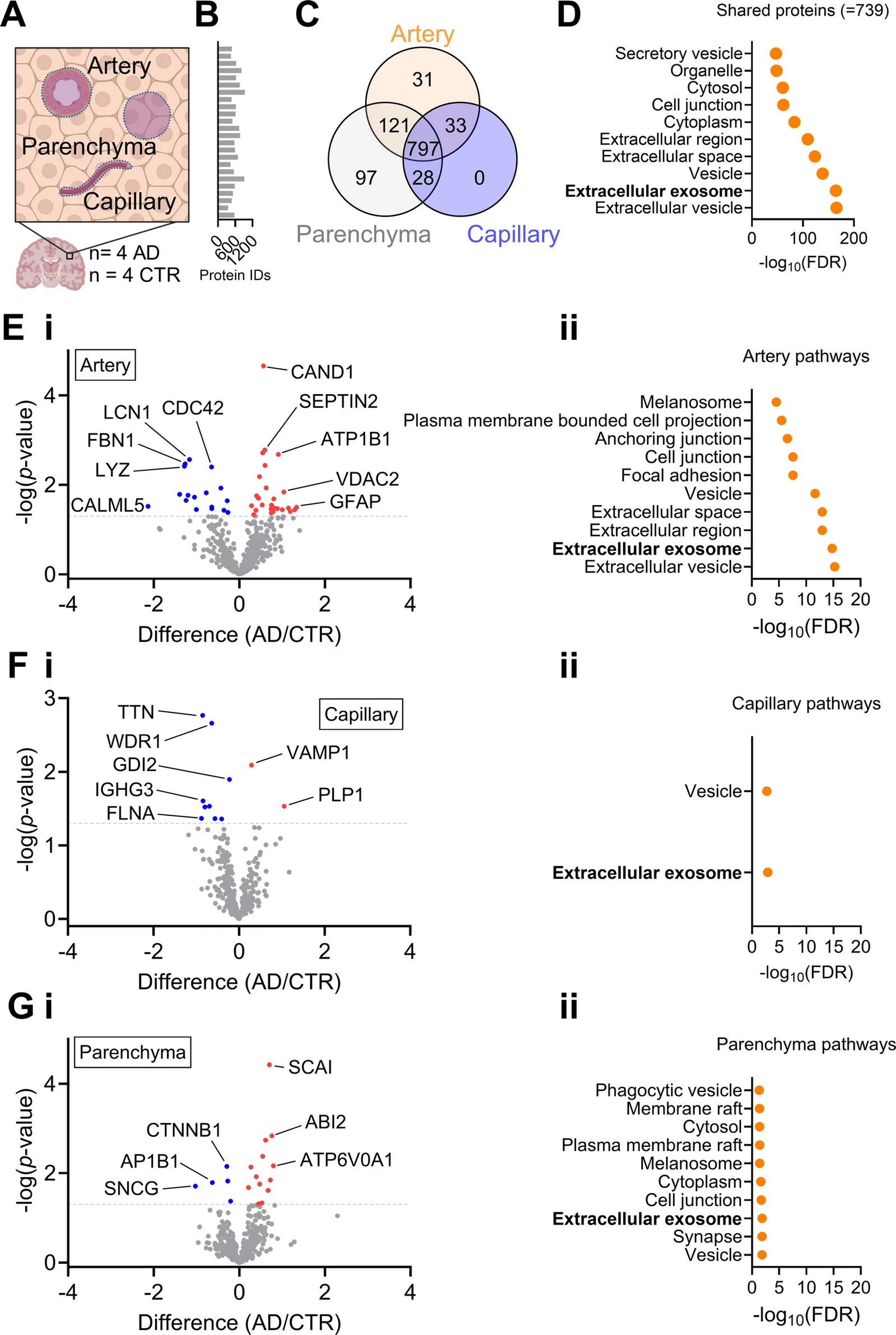
Spatial proteomic analysis cerebrovasculature in Alzheimer’s disease (AD) patients and non-demented controls (CTR). (**A**) Graphical illustration of regions of interests (ROI) in laser microdissection (LMD) setup (artery, capillary and adjacent parenchyma). (**B**) Representation of proteins identified by liquid chromatography–tandem MS (LC–MS/MS) using data-independent acquisition (DIA) of LMD samples. (**C**) Venn diagram showing number of shared proteins identified in the three ROIs (both AD and CTR). (**D**) Gene overrepresentation analysis of shared proteins using the GO: Cellular Component category. Log10 transformed false-discovery rate (FDR) values (using Benjamini–Hochberg procedure) are presented together with annotated ontology term. (Ei), (Fi) and (Gi) volcano plots comparing protein abundance in the three respective ROIs from AD and CTR. Log2 fold change differences and non-adjusted *p*-values are presented. Red = upregulated and blue = downregulated in AD compared to CTR. (Eii), (Fii) and (Gii) Gene overrepresentation analysis using the GO: Cellular Component category as in ‘(**D**)’

The primary effect of hypertension is progressive structural and functional damage to arteries, which has been demonstrated extensively in the SHR model. Based on the shared dysregulation of the exosome protein signature in the brains in our study, we investigated whether such exosome signature was distinctly present in arteries, capillaries and/or parenchyma in human AD brains. We therefore applied an optimized spatial proteomics protocol by combining laser microdissection (LMD) and high-sensitivity proteomics analysis (Fig. 7A). Our analysis identified a total of 1107 proteins, of which 797 proteins were shared between the three region types (Fig. 7B, C). The shared background proteins (= 797) were significantly associated with the ‘extracellular exosome’ pathway (Fig. 7D). In the arteries, differential expression analysis identified 44 DEPs when comparing AD to non-demented controls (Fig. 7Ei). These DEPs were also significantly associated with the ‘extracellular exosome’ pathway (Fig. 6Eii). Similar expression analysis of capillary- and parenchyma-enriched tissue identified 11 and 19 DEPs, respectively, which also associated with the exosome pathway (Fig. 7F, G). However, despite the consistent association to the ‘extracellular exosome’ pathway, the significance varied markedly between the ROIs, with arteries showing a high significance (-log10(FDR) = 14.78), while capillaries and parenchyma showed a moderate significance (-log10(FDR) = 2.89 and 1.85, respectively). This supported a stronger protein representation of the exosome protein signature in cerebral arteries from the AD patients compared to control.

### Proteomic comparison identifies 17 overlapping proteins in 5xFAD model, whose trajectory pattern is shared with AD Braak staging

**Fig. 8:**
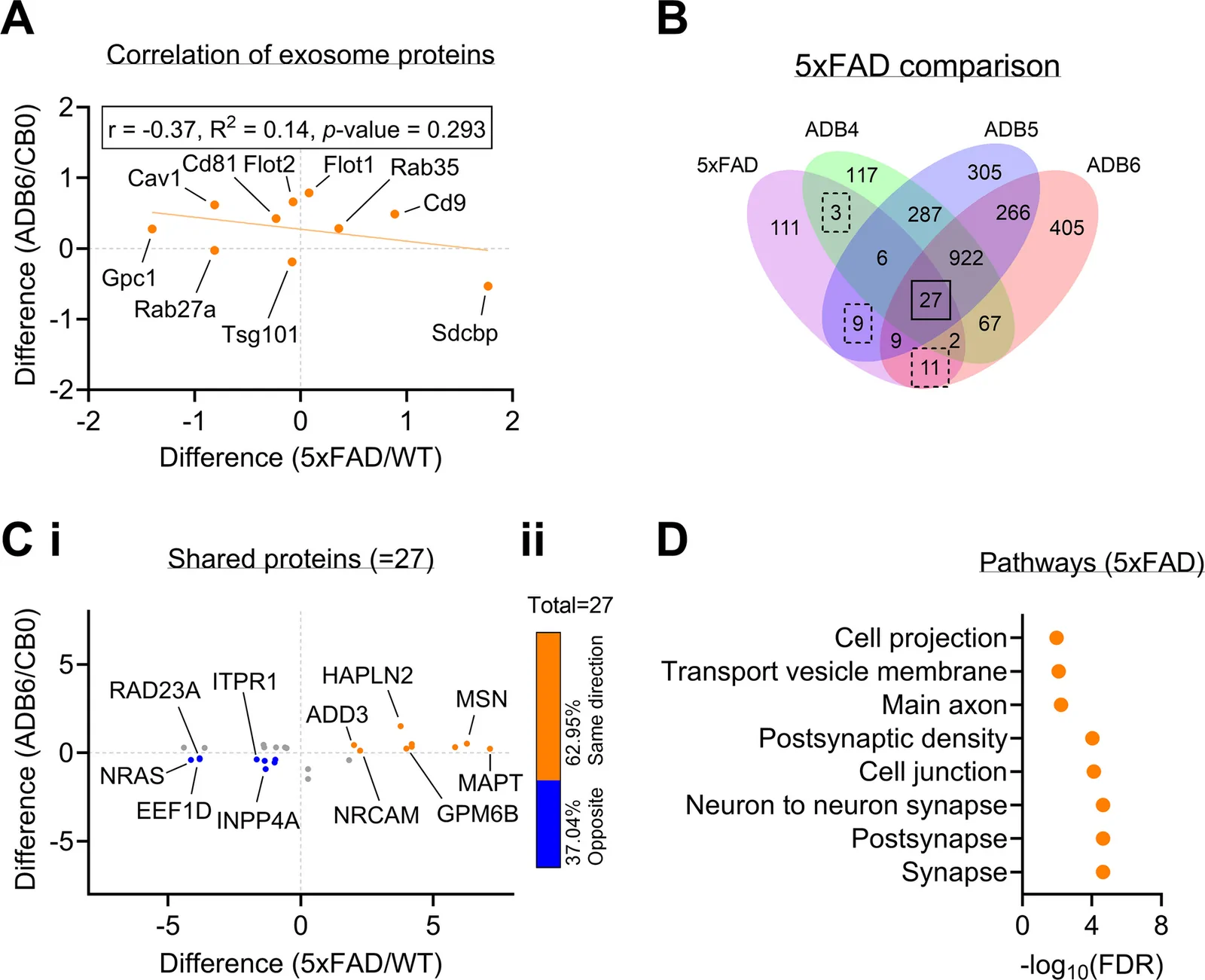
Proteomic comparison to transgenic mouse model of familial Alzheimer’s disease (5xFAD). (**A**) Correlation between fold change differences in exosome marker proteins from human Alzheimer’s disease (AD) Braak 6 brains (ADB6) versus non-demented control Braak 0 (CB0) and dataset from 5xFAD mouse model of familial AD versus wildtype (WT) control [37]. (**B**) Venn-diagram comparing lists of differentially expressed proteins from human (Braak 4–6) and 5xFAD model. (Ci) Scatter plot depicting fold change difference from 5xFAD and ADB6 comparisons. Orange = shared upregulated proteins and blue = shared downregulated proteins. (Cii) Representation of the number of overlapping proteins that regulated in same direction (orange) or opposite direction (blue). (**D**) Gene overrepresentation analysis of shared candidate proteins using the GO: Cellular component category. Log10 transformed false-discovery rate (FDR) values (using Benjamini–Hochberg procedure) are presented together with annotated ontology term

To differentiate vascular versus genetic contributions to AD-like protein signature in the SHR model, we compared the human AD proteome to a similar dataset generated from cortex brain tissue of 6-month-old transgenic 5xFAD mice, a model of familial AD [37]. Focusing on the same exosome protein markers as shown in (Fig. 3), correlation analysis revealed no significant relationship (*p*-value = 0.293) between fold change differences in the AD Braak 6 stage and 5xFAD compared to age-matched wildtype (WT) control mice (Fig. 3A). After differential expression analysis, we identified 189 DEPs when comparing 5xFAD mice to control. When comparing the list of DEPs to those obtained in the human AD Braak stages, we identified 27 overlapping proteins (Fig. 8B). Of these, 17 proteins (67%) were regulated in the same direction (Fig. 8C). We then performed pathway enrichment analysis and observed association with ‘Synapse’, ‘Cell junction’ and ‘Main axon’; however, there was no association with the ‘extracellular exosome’ term (Fig. 8D).

## Discussion

This study provides novel insights into the molecular mechanisms by which untreated hypertension is associated with an AD-like phenotype in the frontal cortex of the SHR model. Herein, we identify changes in several proteins that are associated with exosome protein signatures, which may represent a novel dysregulated pathway in both diseases. Furthermore, we show that the aged SHR better reflects the dysregulated exosome protein signature observed in human AD brains compared to the commonly used 5xFAD model.

Our experimental design allowed us to characterize the protein changes occurring in the brain as a consequence of chronic exposure to high blood pressure in the SHR model together with human brain tissue analysis from AD patients, ranging from Braak stages 0 to 6. To confirm that the SHR had an AD-like phenotype, we used a novel proteomic method to determine the Aβ to sAPP ratio. This analysis was validated in the AD patients, where the Braak 4–6 samples displayed an increased ratio compared to the non-demented Braak 0–3 control brains. In the SHR, there was an overall increase in the Aβ to sAPP ratio compared to the normotensive WKY rat. These changes suggest that the shift towards the Aβ accumulation was due to the difference in blood pressure rather than being age-dependent. The underlying mechanisms causing Aβ accumulation in the SHR need further investigation, although hypotheses have been proposed, e.g. suppression of glymphatic clearance [38] and a bloodstream origin rather than being a neuronal source [39]. Studies have investigated Aβ aggregation and deposition in the SHR and SHR-SP models using immunohistochemistry assays, but reports are contradictory, with some showing increased aggregation [39] and others reporting no difference [40]. These discrepancies may arise from antibody specificity (e.g. targeting APP, Aβ or Aβ fibrils), cross-species reactivity, or the biochemical properties of the rat Aβ1–42. Our proteomic method for measuring the Aβ to sAPP ratio is arguably better suited for comparing Aβ levels as it does not rely on antibodies.

In addition to increased Aβ to sAPP ratio, our proteomic data identified significantly increased levels of total tau (MAPT), both in the SHR compared to WKY controls and in AD patients compared to non-demented controls. However, we did not detect aggregation of pTau using the AT8 antibody-based immunostaining in the SHR model (data not shown). Thus, the SHR model appears indicative of the early stages of late-onset AD, before clinical symptoms develop in humans and before pathological accumulations. Notably, our proteomic comparison of the SHR to the commonly used 5xFAD model of AD revealed a similar number of DEPs overlapping with human AD Braak stages 4–6 (= 17 DEPs in the 5xFAD model and = 24 DEPs in the SHR model). Pathway enrichment analysis confirmed association with the ‘extracellular exosome’ pathway signature in the SHR model, which was absent in the 5xFAD mice. This highlights the SHR as a novel model for capturing aspects of sporadic AD that may not be represented in the commonly used 5xFAD model.

Based on the presence of Aβ dysregulation and potential lack of pTau aggregation (and neurofibrillary tangle (NFT) formation) in the SHR, it could be reasoned that the SHR mimics a CAA phenotype rather than AD. However, our proteomic comparison of DEPs from the SHR revealed a higher number of overlapping proteins with the AD Braak stages compared to CAA, suggesting a closer resemblance of AD-like pathology. As such, this model may offer novel insights into prodromal AD, particularly AD associated with hypertension as a risk factor, thereby advancing research into novel biomarkers and early treatments for late-onset AD.

Our in-depth proteomic analysis across different Braak stages allowed us to perform a pseudo-temporal trajectory of proteomic changes related to the progression of AD pathology. This is supported by a strong clustering and separation of groups in the PCA, suggesting a shared protein expression profile. The identification of over 1000 DEPs in all AD groups highlights the complexity of AD pathogenesis and supports the notion that many protein changes are occurring around the two main hallmarks (Aβ and p-tau) in sporadic AD. Furthermore, the number of DEPs identified in comparisons with the non-demented controls was limited and did not follow a gradual linear relationship (< 200 in control versus > 1000 in AD), supporting a substantial impact on general protein regulation following the manifestation of the two main AD hallmarks in sporadic AD.

Our discovery-based proteomic design identified 24 candidate proteins whose trajectory was mirrored between the pseudo-Braak staging and the SHR model. These candidate proteins were significantly associated with the ‘extracellular exosome’ pathway term. Exosomes are 30–150 nm sized vesicles encasing e.g. proteins, cytokines and miRNA [41]. When fusing with the plasma membrane, exosomes are released into extracellular space for intercellular communication [42]. Importantly, it is known that exosomes are dysregulated in AD and involved in the spreading of Aβ and p-tau [43, 44]. However, the predominant assumption is that exosomes are derived from neurons and glial cells [45]. Our data demonstrates that the cerebrovasculature in AD patients is significantly associated with a dysregulation of the ‘extracellular exosome’ pathway signature. While the presence of CD81 signal, within and around the VSMC layer predominately, supports a vascular origin, the possibility of glial sources, such as astrocytes supporting the BBB, cannot be excluded and warrants further investigation. This was supported by the significant increase of the astrocytic marker, GFAP, in the microdissected arteries from AD patients compared to controls. It has recently been recognized that exosomes have a significant role in the pathophysiology of hypertension and associated vascular remodeling [46–49]. Furthermore, angiogenic dysfunction, which is associated with exosome dysregulation, is a prevalent observation in both AD and hypertension [50–53]. Together with the positive and strong correlation of fold change difference of exosome marker proteins, our data points to a critical dysregulation of exosomes in the cerebrovasculature of both AD patients and the SHR model. The observation that this dysregulation is shared highlights the benefit of using the SHR model to study exosomes and the exosome protein signature in a late-onset AD context. Using this novel model, future studies can now investigate the pathological role of exosomes in a phenotypic model that mirrors a significant risk factor of late onset AD in the absence of familial genetic AD mutations.

Our study has limitations. First, it was not possible to obtain information on blood pressure or anti-hypertensive medication in human samples used for the proteomic analysis (cohort 2), and therefore we cannot ascertain a blood pressure-dependent effect similar to that observed in the SHR model. Second, we did not have access to adjacent FFPE slices of the same human samples used for the proteomic analysis to obtain spatial information on the identified proteins. To solve this, we used a second cohort of AD patients and non-demented controls (cohort 1). We thereby supported the dysregulation of the exosome pathway signature across two independent AD cohorts.

This study has revealed numerous protein changes in the frontal cortex regions of the SHR, as well as the superior and medial frontal gyrus region of AD and CAA brain tissue. The in-depth proteomic profiling has identified candidate proteins with a significant association with an exosome protein signature. These vesicles have been implicated in AD pathogenesis previously, but we are still far from understanding their mechanistic contribution to AD. One reason for this is the lack of good animal models. Our data suggests that the SHR could be a useful animal model for studying the role of exosomes in AD and may aid in the development of novel biomarkers or treatment targets for AD. Finally, this study identified a significant protein overlap in brain tissue from hypertensive rats and human AD, providing a novel mechanistic link into how untreated hypertension is a risk factor for sporadic AD development.

## Supplementary Information

Below is the link to the electronic supplementary material.

ESM 1 (DOCX 2.90 MB)

ESM 2 (XLSX 6.90 MB)

## Data Availability

The mass spectrometry proteomics data have been deposited to the ProteomeXchange Consortium via the PRIDE[34] partner repository with the dataset identifiers PXD057298 and PXD066265.

## References

[1] Serrano-Pozo A, Frosch MP, Masliah E, Hyman BT. Neuropathological alterations in Alzheimer disease. Cold Spring Harb Perspect Med. 2011;1:a006189–a006189. 10.1101/cshperspect.a006189 PMC3234452 22229116

[2] Østergaard L, Aamand R, Gutiérrez-Jiménez E, Ho YCL, Blicher JU, Madsen SM, et al. The capillary dysfunction hypothesis of Alzheimer’s disease. Neurobiol Aging. 2013;34:1018–31. 10.1016/j.neurobiolaging.2012.09.011 23084084

[3] Mattsson N, Tosun D, Insel PS, Simonson A, Jack CR, Beckett LA, et al. Association of brain amyloid-β with cerebral perfusion and structure in Alzheimer’s disease and mild cognitive impairment. Brain. 2014;137:1550–61. 10.1093/brain/awu043 PMC3999717 24625697

[4] Ruitenberg A, den Heijer T, Bakker SLM, van Swieten JC, Koudstaal PJ, Hofman A, et al. Cerebral hypoperfusion and clinical onset of dementia: the Rotterdam study. Ann Neurol. 2005;57:789–94. 10.1002/ana.20493 15929050

[5] Yew B, Nation DA. Cerebrovascular resistance: effects on cognitive decline, cortical atrophy, and progression to dementia. Brain. 2017;140:1987–2001. 10.1093/brain/awx112 PMC6059092 28575149

[6] de la Torre JC, Mussivan T. Can disturbed brain microcirculation cause Alzheimer’s disease? Neurol Res. 1993;15:146–53. 10.1080/01616412.1993.11740127 8103579

[7] de la Torre JC. Is Alzheimer’s disease a neurodegenerative or a vascular disorder? Data, dogma, and dialectics. Lancet Neurol. 2004;3:184–90. 10.1016/S1474-4422(04)00683-0 14980533

[8] Biancardi VC, Son SJ, Ahmadi S, Filosa JA, Stern JE. Circulating angiotensin II gains access to the hypothalamus and brain stem during hypertension via breakdown of the blood–brain barrier. Hypertension. 2014;63:572–9. 10.1161/HYPERTENSIONAHA.113.01743 PMC4080808 24343120

[9] Iadecola C, Gottesman RF. Cerebrovascular alterations in Alzheimer disease. Circ Res. 2018;123:406–8. 10.1161/CIRCRESAHA.118.313400 PMC6214471 30355253

[10] Wu C, Zhou D, Wen C, Zhang L, Como P, Qiao Y. Relationship between blood pressure and Alzheimer’s disease in Linxian County, China. Life Sci. 2003;72:1125–33. 10.1016/s0024-3205(02)02367-6 12505543

[11] Launer LJ, Ross GW, Petrovitch H, Masaki K, Foley D, White LR, et al. Midlife blood pressure and dementia: the Honolulu-Asia aging study☆. Neurobiol Aging. 2000;21:49–55. 10.1016/s0197-4580(00)00096-8 10794848

[12] Joas E, Bäckman K, Gustafson D, Östling S, Waern M, Guo X, et al. Blood pressure trajectories from midlife to late life in relation to dementia in women followed for 37 years. Hypertension. 2012;59:796–801. 10.1161/HYPERTENSIONAHA.111.182204 22331381

[13] Jung H, Yang P-S, Kim D, Jang E, Yu HT, Kim T-H, et al. Associations of hypertension burden on subsequent dementia: a population-based cohort study. Sci Rep. 2021;11:12291. 10.1038/s41598-021-91923-8 PMC8192762 34112942

[14] Ding J, Davis-Plourde KL, Sedaghat S, Tully PJ, Wang W, Phillips C, et al. Antihypertensive medications and risk for incident dementia and Alzheimer’s disease: a meta-analysis of individual participant data from prospective cohort studies. Lancet Neurol. 2020;19:61–70. 10.1016/S1474-4422(19)30393-X PMC7391421 31706889

[15] Scheffer S, Hermkens DMA, van der Weerd L, de Vries HE, Daemen MJAP. Vascular hypothesis of Alzheimer disease. Arterioscler Thromb Vasc Biol. 2021;41:1265–83. 10.1161/ATVBAHA.120.311911 33626911

[16] Drummond E, Wisniewski T. Alzheimer’s disease: experimental models and reality. Acta Neuropathol. 2017;133:155–75. 10.1007/s00401-016-1662-x PMC5253109 28025715

[17] Livingston G, Huntley J, Sommerlad A, Ames D, Ballard C, Banerjee S, et al. Dementia prevention, intervention, and care: 2020 report of the Lancet Commission. Lancet. 2020;396:413–46. 10.1016/S0140-6736(20)30367-6 PMC7392084 32738937

[18] Jandke S, Garz C, Schwanke D, Sendtner M, Heinze H-J, Carare RO, et al. The association between hypertensive arteriopathy and cerebral amyloid angiopathy in spontaneously hypertensive stroke-prone rats. Brain Pathol. 2018;28:844–59. 10.1111/bpa.12629 PMC8028507 30062722

[19] Schreiber S, Drukarch B, Garz C, Niklass S, Stanaszek L, Kropf S, et al. Interplay between age, cerebral small vessel disease, parenchymal amyloid-β, and tau pathology: longitudinal studies in hypertensive stroke-prone rats. J Alzheimers Dis. 2014;42:S205–15. 10.3233/JAD-132618 24825568

[20] Okamoto K, Aoki K. Development of a strain of spontaneously hypertensive rats. Jpn Circ J. 1963;27:282–93. 10.1253/jcj.27.282 13939773

[21] Zhou J, Yu J-T, Wang H-F, Meng X-F, Tan C-C, Wang J, et al. Association between stroke and Alzheimer’s disease: systematic review and meta-analysis. J Alzheimers Dis. 2014;43:479–89. 10.3233/JAD-140666 25096624

[22] Tayebati SK, Tomassoni D, Amenta F. Spontaneously hypertensive rat as a model of vascular brain disorder: microanatomy, neurochemistry and behavior. J Neurol Sci. 2012;322:241–9. 10.1016/j.jns.2012.05.047 22726353

[23] Yamagata K, Tagami M, Yamori Y. Neuronal vulnerability of stroke-prone spontaneously hypertensive rats to ischemia and its prevention with antioxidants such as vitamin E. Neuroscience. 2010;170:1–7. 10.1016/j.neuroscience.2010.07.013 20633610

[24] Johnson AC, Miller JE, Cipolla MJ. Memory impairment in spontaneously hypertensive rats is associated with hippocampal hypoperfusion and hippocampal vascular dysfunction. J Cereb Blood Flow Metab. 2020;40:845–59. 10.1177/0271678X19848510 PMC7168795 31088235

[25] Lolansen SD, Barbuskaite D, Ye F, Xiang J, Keep RF, MacAulay N. Spontaneously hypertensive rats can become hydrocephalic despite undisturbed secretion and drainage of cerebrospinal fluid. Fluids Barriers CNS. 2023;20:53. 10.1186/s12987-023-00448-x PMC10318838 37403103

[26] Bastrup JA, Jepps TA. Proteomic mapping reveals dysregulated angiogenesis in the cerebral arteries of rats with early-onset hypertension. J Biol Chem. 2023;299(10): 105221. 10.1016/j.jbc.2023.105221. PMC10558802 37660920

[27] Bastrup JA, Rippe C, Swärd K, Jepps TA. Temporal proteomic remodeling of cerebral arteries in male hypertensive rats. Hypertension. 2026;83(1):73–86. 10.1161/HYPERTENSIONAHA.125.26119. PMC12711280 41221587

[28] Bastrup JA, Aalkjær C, Jepps TA. Identification of novel proteins and mechanistic pathways associated with early-onset hypertension by deep proteomic mapping of resistance arteries. J Biol Chem. 2022;298:101512. 10.1016/j.jbc.2021.101512 PMC8760518 34929167

[29] Wiśniewski JR, Zougman A, Nagaraj N, Mann M. Universal sample preparation method for proteome analysis. Nat Methods. 2009;6:359–62. 10.1038/nmeth.1322 19377485

[30] Demichev V, Messner CB, Vernardis SI, Lilley KS, Ralser M. Dia-nn: neural networks and interference correction enable deep proteome coverage in high throughput. Nat Methods. 2020;17:41–4. 10.1038/s41592-019-0638-x PMC6949130 31768060

[31] Tyanova S, Temu T, Sinitcyn P, Carlson A, Hein MY, Geiger T, et al. The perseus computational platform for comprehensive analysis of (prote)omics data. Nat Methods. 2016;13:731–40. 10.1038/nmeth.3901 27348712

[32] Szklarczyk D, Franceschini A, Wyder S, Forslund K, Heller D, Huerta-Cepas J, et al. STRING v10: protein–protein interaction networks, integrated over the tree of life. Nucleic Acids Res. 2015;43:D447–52. 10.1093/nar/gku1003 PMC4383874 25352553

[33] Shannon P, Markiel A, Ozier O, Baliga NS, Wang JT, Ramage D, et al. Cytoscape: a software environment for integrated models of biomolecular interaction networks. Genome Res. 2003;13:2498–504. 10.1101/gr.1239303 PMC403769 14597658

[34] Perez-Riverol Y, Csordas A, Bai J, Bernal-Llinares M, Hewapathirana S, Kundu DJ, et al. The PRIDE database and related tools and resources in 2019: improving support for quantification data. Nucleic Acids Res. 2019;47:D442–50. 10.1093/nar/gky1106 PMC6323896 30395289

[35] Askenazi M, Kavanagh T, Pires G, Ueberheide B, Wisniewski T, Drummond E. Compilation of reported protein changes in the brain in Alzheimer’s disease. Nat Commun. 2023;14:4466. 10.1038/s41467-023-40208-x PMC10368642 37491476

[36] Bi R, Zhang W, Zhang D-F, Xu M, Fan Y, Hu Q-X, et al. Genetic association of the cytochrome c oxidase-related genes with Alzheimer’s disease in Han Chinese. Neuropsychopharmacology. 2018;43:2264–76. 10.1038/s41386-018-0144-3 PMC6135758 30054583

[37] Lee S, Jang K, Lee H, Jo YS, Kwon D, Park G, et al. Multi-proteomic analyses of 5xFAD mice reveal new molecular signatures of early-stage Alzheimer’s disease. Aging Cell. 2024;23:1–17. 10.1111/acel.14137 PMC11166370 38436501

[38] Mortensen KN, Sanggaard S, Mestre H, Lee H, Kostrikov S, Xavier ALR, et al. Impaired glymphatic transport in spontaneously hypertensive rats. J Neurosci. 2019;39:6365–77. 10.1523/JNEUROSCI.1974-18.2019 PMC6687896 31209176

[39] Wang Y, Zhang R, Tao C, Xu Z, Chen W, Wang C, et al. Blood-brain barrier disruption and perivascular beta-amyloid accumulation in the brain of aged rats with spontaneous hypertension: evaluation with dynamic contrast-enhanced magnetic resonance imaging. Korean J Radiol. 2018;19:498. 10.3348/kjr.2018.19.3.498 PMC5904477 29713228

[40] Denver P, D’adamo H, Hu S, Zuo X, Zhu C, Okuma C, et al. A novel model of mixed vascular dementia incorporating hypertension in a rat model of Alzheimer’s disease. Front Physiol. 2019;10:1–29. 10.3389/fphys.2019.01269 PMC6821690 31708792

[41] Sardar Sinha M, Ansell-Schultz A, Civitelli L, Hildesjö C, Larsson M, Lannfelt L, et al. Alzheimer’s disease pathology propagation by exosomes containing toxic amyloid-beta oligomers. Acta Neuropathol. 2018;136:41–56. 10.1007/s00401-018-1868-1 PMC6015111 29934873

[42] Xu M, Ji J, Jin D, Wu Y, Wu T, Lin R, et al. The biogenesis and secretion of exosomes and multivesicular bodies (MVBs): intercellular shuttles and implications in human diseases. Genes Dis. 2023;10:1894–907. 10.1016/j.gendis.2022.03.021 PMC10363595 37492712

[43] Weng S, Lai Q-L, Wang J, Zhuang L, Cheng L, Mo Y, et al. The role of exosomes as mediators of neuroinflammation in the pathogenesis and treatment of Alzheimer’s disease. Front Aging Neurosci. 2022;14:1–11. 10.3389/fnagi.2022.899944 PMC9273880 35837481

[44] Yuyama K, Igarashi Y. Exosomes as carriers of Alzheimer’s amyloid-ß. Front Neurosci. 2017;11:1–5. 10.3389/fnins.2017.00229 PMC5403946 28487629

[45] Badhwar A, Haqqani AS. Biomarker potential of brain-secreted extracellular vesicles in blood in Alzheimer’s disease. Alzheimers Dement Diagn Assess Dis Monit. 2020;12:1–14. 10.1002/dad2.12001 PMC7085285 32211497

[46] Arishe OO, Priviero F, Wilczynski SA, Webb RC. Exosomes as intercellular messengers in hypertension. Int J Mol Sci. 2021;22:11685. 10.3390/ijms222111685 PMC8583750 34769116

[47] Su S-A, Xie Y, Fu Z, Wang Y, Wang J-A, Xiang M. Emerging role of exosome-mediated intercellular communication in vascular remodeling. Oncotarget. 2017;8:25700–12. 10.18632/oncotarget.14878 PMC5421963 28147325

[48] Liu X, Yuan W, Yang L, Li J, Cai J. MiRNA profiling of exosomes from spontaneous hypertensive rats using next-generation sequencing. J Cardiovasc Transl Res. 2019;12:75–83. 10.1007/s12265-017-9784-7 29557993

[49] Perez-Hernandez J, Riffo-Campos AL, Ortega A, Martinez-Arroyo O, Perez-Gil D, Olivares D, et al. Urinary- and plasma-derived exosomes reveal a distinct microRNA signature associated with albuminuria in hypertension. Hypertension. 2021;77:960–71. 10.1161/HYPERTENSIONAHA.120.16598 33486986

[50] Akbarian M, Bertassoni LE, Tayebi L. Biological aspects in controlling angiogenesis: current progress. Cell Mol Life Sci. 2022;79:349. 10.1007/s00018-022-04348-5 PMC10171722 35672585

[51] Tarawneh R. Microvascular contributions to Alzheimer disease pathogenesis: is Alzheimer disease primarily an endotheliopathy? Biomolecules. 2023;13:830. 10.3390/biom13050830 PMC10216678 37238700

[52] Govindpani K, McNamara LG, Smith NR, Vinnakota C, Waldvogel HJ, Faull RLM, et al. Vascular dysfunction in Alzheimer’s disease: a prelude to the pathological process or a consequence of it? J Clin Med. 2019;8:1–57. 10.3390/jcm8050651 PMC6571853 31083442

[53] Govindpani K, Vinnakota C, Waldvogel H, Faull R, Kwakowsky A. Vascular dysfunction in Alzheimer’s disease: a biomarker of disease progression and a potential therapeutic target. Neural Regen Res. 2020;15:1030. 10.4103/1673-5374.270306 PMC7034279 31823877

