# Supplementary material for: Extracellular exosome signature links hypertension and Alzheimer’s disease
Source: GeroScience. 2026 Feb 4;48(6):8265–83. doi: 10.1007/s11357-026-02097-y (PMC13638816; doi:10.1007/s11357-026-02097-y)
Supplement: Supplementary file 1 [file 11357_2026_2097_MOESM1_ESM.docx]

Supplementary Information for

**Extracellular Exosome Signature Links Hypertension and Alzheimer’s Disease.**

Joakim A. Bastrup^1^*, Jonas Folke^2^, Sanne S Kaalund^2^, Michel Rasmussen^2^, Susana Aznar^2^, Thomas Jepps^1^.

Authors’ affiliation: ^1^Vascular Biology Group, Department of Biomedical Sciences, Faculty of Health and Medical Sciences, University of Copenhagen, Copenhagen, Denmark.

^2^Centre for Neuroscience and Stereology, Department of Neurology, Bispebjerg and Frederiksberg Hospital, Copenhagen University Hospital, Copenhagen, Denmark.

*Corresponding Author: Joakim A. Bastrup. Department of Biomedical Sciences, Faculty of Health and Medical Sciences, University of Copenhagen, Copenhagen, Denmark.

Email: [joakim.bastrup@sund.ku.dk](mailto:joakim.bastrup@sund.ku.dk)

Telephone: +45 31137569

**This file includes:**

Supporting text: Methods.

Figures S1 to S4

Supporting Tables S1 and S3 (Supplementary Table S2 is attached separately as an Excel file)

Supporting materials References

**Supporting text: Methods.**

*Experimental animals*

The animal experiments with SHR and Wistar Kyoto (WKY) rats were approved by local Animal Care and Use Committees (institutional approval numbers P23-177 and P24-062). The experiments were conducted in accordance with the directives of the Danish National Committee on Animal Research Ethics and Danish legislation on experimental animals. The rats were purchased from Janvier Labs and arrived at 10 weeks of age and group housed (3-4 rats per cage) until the end of the study. All rats were kept in a temperature-controlled environment with a 12/12-h light–dark cycle and had free access to standard chow and tap water ad libitum. Clean cages were provided once a week.

Anaesthesia was induced by 5 % isoflurane delivered in a mixture of 65 % nitrogen and 35 % oxygen, and rats at 30 or 40 weeks of age were euthanized by decapitation while still under anaesthesia. The major cerebral arteries from these rats were also used in previous study by Bastrup et al. (2025)[1]. In a subgroup of animals (n=2 SHRs and WKYs) from the same cohorts, the left carotid artery was catheterized for arterial blood pressure measurement. In brief, after anaesthesia induction, the rats were intubated, connected to a respirator (tidal volume 8 ml/kg bw; frequency 69 breaths/min), and placed on a heating table to maintain a constant body temperature (37 °C). The left carotid artery was cannulated with a catheter connected to a pressure transducer (Statham P23-dB) for continuous monitoring of the blood pressure monitoring. A final isoflurane concentration of 1 %–2 % maintained sufficient anaesthesia. Increased mean blood pressure over a stable 3 min period was confirmed in SHRs compared with WKYs (Mean blood pressure ± standard deviation: SHR = 157.8 ± 12.6, WKY = 99.1 ± 8.3). After the stable recording, the rats were euthanized by decapitation[2].

The brains were carefully excised, rinsed in cold physiological salt solution (121 mM NaCl; 2.8 mM KCl; 1.2 mM KH_2_PO_4_; 1.2 mM MgSO_4_; 25 mM NaHCO_3_; 1.6 mM CaCl_2_; 0.03 mM EDTA; 5.5 mM D-glucose) saturated with carbogen (O_2_ 95 %; CO_2_ 5 %) at pH 7.4. The brains were then divided into two hemispheres and snap frozen in liquid nitrogen or incubated in 10 % neutral buffered formalin followed by storage at -80 °C or 4 °C, respectively.

*Demographics of human samples*

The human brains analyzed in the current study came from two cohorts. The first cohort was donated to the Bispebjerg Brain Bank at Bispebjerg-Frederiksberg Hospital (Copenhagen University Hospital, Denmark), and the second to the Netherlands Brain Bank for later inclusion at Bispebjerg Brain Bank. The donated brains from both cohorts were neuropathologically examined to verify the diagnosis. Cohort 1 included brain samples from 4 brains with AD pathology and 4 brains with mild primary age-related tau (PART) pathology (Braak stage 1-3, Thal phase 0-1) from non-demented individuals. AD pathology was defined as the presence of neuronal neurofibrillary tangles and amyloid beta positive plaques exceeding Braak neurofibrillary stage 4 and Thal phase 3, respectively[3]. Neuritic plaques were not assessed. Amyloid angiopathy affecting the capillaries only were seen in one of the cases with AD pathology, while amyloid angiopathy affecting both capillaries and the meningial arteries was observed in two of the AD pathology brains. In two brains, AD pathology was found as concomitant pathology to either corticobasal degeneration, a 4R tauopathy, or multiple system astrophy (a synucleopathy with glial cytoplasmic inclusions of alpha-synuclein).

Cohort 2 included 30 patients diagnosed with AD (ranging from Braak stages 4-6), 5 patients diagnosed with cerebral amyloid angiopathy (CAA), and 37 brain samples from non-demented individuals (ranging from Braak stages 0-3). The collection and analysis of the samples were conducted in accordance with the World Medical Association Declaration of Helsinki and approved by the regional ethical committee of the Capital Region (Region Hovedstaden, Denmark), j.nr. H-23030276, and the Danish Data Protection Agency (j.nr. p-2023-15234). All donors provided informed and written consent. Brain tissue was stored at −80 °C until protein extraction.

*Statistical analysis of demographics*

Statistical analyses were conducted to assess differences in demographic characteristics across the cohorts and subgroups (Table 1; SI Table 2). The variables analyzed included age, sex, postmortem interval (PMI) and APOE genotype. Descriptive statistics are presented as mean (standard deviation) and range for continuous variables. Normality was assessed using D’Agostino & Pearson test. Age and PMI was analyzed using Kruskal-Wallis followed by Dunn’s test for multiple comparison. Sex and APOE genotypes were analyzed using chi-squared test. Specifically, significant differences were observed in age between CB3 and CB0 (KW: p = 0.029; Dunn’s: p = 0.031). A significant difference in PMI was observed (KW: p = 0.036); however, no significance was found between groups. There were no significant differences in sex distribution (χ^2^: p = 0.552) or APOE genotype (χ^2^: p = 0.162).

*Sample preparation*

Tissues (approximately 0.3 cm × 0.3 cm × 0.3 cm) were homogenized in 300 μl of ice-cold lysis buffer (50 mM Tris pH 8.5; 5 mM EDTA pH 8.0; 150 mM NaCl; 10 mM KCl; 1 % NP-40; and 1X complete protease inhibitor cocktail (Roche, cat no. 04693124001)) by three rounds of chopping the tissue using dissection scissors and a handheld homogenizer[4]. The homogenates were centrifuged at 11,000 g for 10 min at 4 °C to obtain the supernatant. Protein quantification of the tissue extracts was determined by bicinchoninic acid (BCA) assay (Thermo Fisher Scientific, cat no. 23227).

Tissue extracts (50 μg protein) were diluted in digestion buffer containing 0.5 % sodium deoxycholate in 50 mM triethylammonium bicarbonate and heated to 70 °C for 10 min. The samples were then cooled on ice and prepared using filter-aided sample preparation (FASP) [5] with flat spin filters (Microcon-10 kDa, Cat no. MRCPRT010). The samples were reduced and alkylated in digestion buffer containing 1:50 (v:v) tris(2-carboxyethyl)phosphine (0.5 M; Sigma, Cat no. 646547) and 1:10 (v:v) 2-chloroacetamide (0.5 M; Sigma, Cat no. C0267) for 30 min at 37 °C. Proteolytic digestion was performed overnight at 37 °C by addition of 0.5 μg Trypsin/LysC mix (Promega, cat no. V5072) (at enzyme to protein ratio 1:100). Centrifugation at 14,000 g for 15 min at 4 °C was employed between the different steps. The resulting peptides were desalted using stage-tips containing a Poly-styrene-divinylbenzene copolymer modified with sulfonic acid groups (SDB-RPS; 3M) material, as previously described [6]. The eluted peptide mixture was dried down using SpeedVac vacuum centrifugation and stored at -80 °C until further analysis.

*Sample preparation for laser microdissection*

Polyethylene naphthalate (PEN) membrane slides (Applied Biosystems^TM^, LCM0522) were coated with Vectabond (Vector Laboratories, Cat. No. SP-1800-7) following the manufacturer’s instructions. FFPE tissue sections were mounted onto pre-treated slides and dried at RT. Slides were deparaffinized, rehydrated and subjected to antigen retrieval as described in the *Histology* section. Sections were stained with anti-alpha smooth muscle actin antibody [1A4] (ab7817, produced in Mouse, 1:400, Abcam) for 60 min at RT, washed twice in 1× PBS for 5 min, and incubated in 3 % H_2_O_2_ (Region H Pharmacy, #221378) for 8 min. Counterstaining was made with anti-mouse HRP-conjugated EnVision+ detection reagent (DAKO, Cat. No. K4001) for 30 minutes at RT followed by two washes in 1× PBS for 5 min. Signal development was performed using the 3,3′-diaminobenzidine (DAB) Bright system (ImmunoLogic, Cat. No. VWEKBS04-500) for 8 minutes. Slides were then rinsed in distilled water, stained with Mayer’s hematoxylin (Region H Pharmacy, Cat. No. 860213) for 30 seconds, wash under running tap water for 7 min and rinsed in deionized water for 3 min. The membrane was punctured by creating small holes and dried for 45 min at 60 °C followed by storage at RT until laser dissection.

Regions of interest (artery, capillary or adjacent parenchyma) were cut using the LMD7 (Leica) laser microdissection system with the following settings: Power = 30, Aperture = 15, Speed = 20, Specimen balance = 15, Head current = 100 %, Puls frequency = 120. The microscope was operated with the LMD v8.2 software, with ThreeSlides sample holder and the standard PCR tube collector (4 tubes). A total tissue area of approximately 200.000 µm^2^ were collected per region into a 0.2 ml thin walled and flat capped PCR tube (Axygen, PCR-02-C). One protein rich ‘booster’ sample containing a tissue area of approximately 1.000.000 µm^2^ was also collected in a separate tube. The dissected elements were confirmed by visual inspection under microscope. Tubes were centrifuged at 5000 g for 10 min, then 25 ul of digestion buffer was added to the cap and tubes were centrifuged again. Tubes were heated for 1 hour at 95 °C in a heat block, cooled to RT and centrifuged for 30 sec at 5000 g. Samples were reduced and alkylated by adding 0.5 ul TCEP (0.5 M, Sigma) and 2.5 ul CAA (0.5 M, Sigma) followed by 20 min incubation at 37 °C. Subsequently, 20 ng of Trypsin/LysC mix (Promega, cat no. V5072) was added and proteins were digested overnight at 37 °C. Samples were desalted as described in the *Sample preparation for proteomics* section and subsequently reconstituted in 7 ul reconstitution buffer.

*Data acquisition by mass spectrometry*

Peptide mixtures (1 and 5 µl injections of rat and human samples, respectively) were analyzed on Bruker timsTOF mass spectrometer (Bruker Daltonics) using positive ion mode, with a Captivespray ion source on-line connected to a Dionex Ultimate 3000RSnano chromatography system (Thermo Fisher). Peptides were separated on an Aurora column with captive spray insert (C18 1.6 uM particles, 15 cm, 75 μm inner diameter; IonOpticks) at 60 °C. A solvent gradient of buffer A (0.1 % formic acid in H_2_O) and buffer B (99.9 % acetonitrile/0.1 % formic acid) was used over 22 min for human samples and 28 min for rat samples, at a flow rate of 400 nL/min and 600 nL/min for human and rat samples, respectively. The mass spectrometer was operated in DIA PASEF mode with 1.1 s cycle time and TIMS ramp time of 100.0 ms. MS scan range was set to 100–1700 m/z. For LMD samples (5 µl injections), an 8 cm Aurora column with captive spray insert (C18 1.7 uM particles, 75 μm inner diameter; IonOpticks) was used together with a 15 min solvent gradient of buffer A and B. The ‘high sensitivity’ was enabled in the TimsControl software for LMD samples.

*Protein identification by mass spectrometry*

Raw DIA files were analyzed with DIA-NN software (v.1.8.1) separately (<https://github.com/vdemichev/diann>) [7]. An in silico DIA-NN predicted spectral library was generated using UniProt FASTA databases for *Rattus norvegicus*; (UP000002494_10116.fasta and UP000002494_10116_additional.fasta, downloaded August 2020), and for *Homo sapiens*; (UP000005640_9606 and UP000005640_9606_additional, downloaded April 2022). The DIA-NN search included the following settings: ‘FASTA digest for library-free search/library generation’ and ‘Deep learning-based spectra, RTs, and IMs prediction’ were enabled, Digestion protease = Trypsin/P; missed cleavage = 1; max number of variable modifications = 1; methionine oxidation (UniMod:35) was considered as variable modification; N-term M excision = True; C carbamidomethylation = True; peptide length range = 7 to 30; precursor charge range = 1 to 4; precursor m/z range = 300 to 1800; fragment ion m/z range = 200 to 1800; Precursor false discovery rate (FDR) (%) = 1 %; Use isotopologs = enabled; MBR = enabled; Neural network classifier = single-pass mode; Protein inference = Genes; Quantification strategy = Any LC (high accuracy); Cross-run normalization = RT-dependent; Library generation = Smart profiling; Speed and RAM usage = Optimal results.

Raw files from LMD samples were analyzed with DIA-NN software (v.1.9) with an in-silico DIA-NN predicted spectral library generated using the human UniProt FASTA database (UP000005640_9606). The same settings were used in DIA-NN, except methionine oxidation (UniMod:35) was not included.

*Bioinformatics*

Protein quantities from brain homogenate samples were obtained from the unique gene list in DIA-NN (report.unique_genes_matrix.tsv) and analyzed using Perseus (v1.6.14.0)[8]. The data was log2 transformed and filtered for by minimum three valid values in each group.

A Two-sided Student’s t-test was conducted with 250 randomizations, permutated FDR < 0.05 and p-value < 0.05. Volcano plots were generated in GraphPad Prism (v10) using uncorrected log-transformed *p-*values and protein differences measured in the t-test. Missing values were only imputed for principal component analysis using width 0.4 and downshift 1.8.

Enrichment analysis of differentially expressed proteins identified from the SHR *versus* WKY comparisons and overlapping candidate proteins was performed with STRING app (v2.0.1)[9] in Cytoscape (v3.9.1)[10]. *Rattus norvegicus* or *Homo sapiens* were selected as species in the analysis, and *genome* was used as background for functional enrichment. The GO: Biological process and GO: Cellular Component (species = homo sapiens) categories were used. Benjamini–Hochberg procedure was used for FDR correction. Confidence score cutoff of 0.3 was used for the PPI network generation.

For cellular deconvolution analysis, data was exported from Perseus into R using the PerseusR package. Cell-type specific gene lists (Total elevated) corresponding to astrocytes (=1251 genes), endothelial (=349 genes), excitatory neuron (=2254 genes), macrophages (=503 genes), microglial (=806 genes), oligodendrocyte (=1630 genes), and smooth muscle cell (=375 genes) were downloaded from <https://www.proteinatlas.org/humanproteome/single+cell/single+cell+type/> and matched to the datasets. Violin plots with overlaid mean intensity bar were generated using ggplot2.

For AD database comparison, the ‘Supplementary data 1 – NeuroPro Dataset’ sheet from the ‘41467_2023_40208_MOESM4_ESM.xlsx’ file was downloaded[11]. Data was converted to .txt and imported into Perseus for further analysis and comparison.

Supplementary data (acel14137-sup-0002-files1.xlsx) was downloaded from previous publication[12] and data from ‘6M_Cortex_WT-TG’ was converted to .txt and imported into Perseus. SynGO (<https://www.syngoportal.org/convert>) was used to retrieve human orthologs from mouse gene symbols[13]. In Perseus, data was filtered based on *p* value < 0.05 and Venn diagram function was used for comparison.

LMD samples were analyzed similarly in Perseus using the protein quantities were obtained from the unique gene list in DIA-NN. The data was log2 transformed and filtered for by minimum three valid values within each region of interest. Data imputation was not used. Two-sided Student’s t-test was used as described above for differential expression analysis.

*Amyloid beta ratio analysis*

Peptide quantities were obtained using the report.pr_matrix.tsv file generated in DIA-NN. For both human and rat samples, all precursors having +2 charge state were used for the analysis. For human samples, the following precursors were selected as full-length APP peptides: ‘AVIQHFQEK’, ‘CAPFFYGGCGGNR’, ‘CLVGEFVSDALLVPDK’, ‘HVFNMLK’, ‘ISYGNDALMPSLTETK’, ‘MDVCETHLHWHTVAK’, ‘QQLVETHMAR’, ‘STNLHDYGMLLPCGIDK’, ‘THPHFVIPYR’, ‘VESLEQEAANER’, ‘WYFDVTEGK‘, and ‘LVFFAEDVGSNK’ was selected as the amyloid core peptide (protein group: P05067). For rat samples, the following precursors were selected as full-length App peptides: ‘AVIQHFQEK’, ‘CAPFFYGGCGGNR’, ‘CLVGEFVSDALLVPDK’, ‘CLVGEFVSDALLVPDKCK’, ‘ISYGNDALMPSLTETK’, ‘KAVIQHFQEK’, ‘MDVCETHLHWHTVAK’, ‘QQLVETHMAR’, ‘SQVMTHLR’, ‘THTHIVIPYR’, ‘VEAMLNDR’, ‘VESLEQEAANER’, ‘WYFDVTEGK’ and ‘MDAEFGHDSGFEVR’ was selected as the amyloid core peptide (protein group: P08592). Quantification in rat samples focused on the amyloid beta core peptide ‘DAEFGHDSGFEVR’, which included an N-terminal methionine (M) in the theoretical digest sequence. The peptides were chosen based on their consistent detection and same charge state across the samples. The peptide ratio was calculated by dividing the intensity of the amyloid beta core peptide by that of the average full-length peptide intensity. Ratios were subsequently transformed using the formula: 1 – (-log_10_(ratio)). Statistical analysis was performed using GraphPad Prism (v10) with One-way ANOVA with Dunnett’s multiple comparisons test for human data, and Two-way ANOVA with Fisher’s LSD test for rat data.

*Histology*

Both rat and human samples were fixed in 10 % neutral buffered formalin for 48 hours and then embedded in paraffin on a Leica ASP300S tissue processor (Leica Biosystems). For rat brain tissue, coronal sections containing hippocampus were cut at a thickness of 16 µm, mounted to Superfrost Plus glass slides, and stored at RT. Human brain tissue was processed similarly, with sections from the occipital cortex. In brief, sections were deparaffinized by sequential washes in xylene (2 x 5 min), followed by a graded ethanol series: 99% ethanol (EtOH, 2 x 2.5 min), 96 % EtOH (2 min) and 70 % EtOH (2 min). Sections were then rinsed in running water for 5 min. Antigen retrieval was performed in citrate-buffer (pH 6; Region H Pharmacy) using a microwave at full power until boiling, then maintained at 30 % power for 15 min. After cooling for 20 min at RT, the sections were rinsed in 1 × PBS for 2 min. Tissue sections were blocked for 30 min in blocking buffer (5 % normal swine serum (Jackson ImmunoResearch, cat no. 014-000-121), 1 % bovine serum albumin (BSA; Sigma, Cat no. A9647), 0.1 % TritonX-100 (Sigma, Cat no. T8787) in 1 × PBS) and stained with commercial anti-alpha smooth muscle actin antibody [1A4] (ab7817, produced in Mouse, 1:400, Abcam), anti-CD81 (HPA007234, produced in Rabbit, 1:400, Sigma) and anti-CD31 (AF3628, produced in goat, 1:100 from 1 µg/µl stock 1× PBS, R&D Systems) diluted in diluted in 1 % BSA, 0.1 % TritonX-100 in 1× PBS overnight at 4 °C. Sections were counterstained with commercial anti-mouse, anti-rabbit, and anti-goat secondary antibodies cross-absorbed to Alexa Flour 488, 564, and 647, respectively, (1:400, Invitrogen) diluted in 1 % BSA, 0.1 % TritonX-100 in 1× PBS for 1 hour at RT. Washes were made 1× PBS or 1× PBS containing 0.5 % Tween after antibody staining. Nuclei were stained with Hoechst 33342 (1:1000 in 1×PBS, Invitrogen) was used after the secondary antibody staining, and sections were mounted in anti-fade mounting medium (Invitrogen, Cat no. S36967).

Fluorescent images were acquired on a LSM900 confocal imaging setup (Zeiss) with a 20×/0.8 Plan-Apochromat Air objective lens (Zeiss). Arteries of interest were selected based on the cross-section morphological quality, presence of all fluorescent markers, and cross-sectional area of approximately 700 µm^2^. Arithmetic mean intensities in regions of interest were measured blinded in ZEN software (v3.2, blue edition, Zeiss). Statistical analyses were made in GraphPad Prism (v10) using unpaired Student’s t-test.

*Western blot*

Isolated protein from rat brains was dissolved in SDS-sample buffer (NuPAGE LDS Sample Buffer (4×), Thermo Scientific, Ref no. NP0007) containing 0.1 M DTT (NuPAGE Sample Reducing Agent (10×), Thermo Scientific, Ref no. NP0009) and heat-treated for 10 min at 70 °C. Proteins (15 µg per sample) were separated using gel electrophoresis on 4-12 % Bis-Tris SDS-PAGE gels (Invitrogen, Cat no. NP0322BOX) and transferred onto polyvinylidene difluoride (PVDF) nitrocellulose membranes (Immobilon-FL, Millipore, Cat no. IPFL00010). After blocking the membranes with Odyssey Blocking buffer (Li-Cor Biosciences, Cat no. 927-70001), they were incubated with primary antibodies anti-Flot1 (HPA001393, 1:1000, Sigma), anti-alpha smooth muscle cell actin (ab32575, 1:2500, Abcam), or anti-CD81 (HPA007234, 1:500, Sigma). The membranes were washed (PBS-Tween 0.1 %) and incubated with conjugated secondary antibodies (α-Rabbit 680 or 800, Li-Cor Biosciences; 1:10,000, respectively) for 1 hour at RT. The Odyssey Infrared Imaging System (Li-Cor Biosciences) was used for protein visualization and supplier’s software (Image Studio Lite, v5.2.5) was used for analysis. Protein bands corresponding to CD81 and Flot1 were normalized to the α-actin band. GraphPad Prism (v10) was used for statistical analysis using unpaired Student’s t-test.

*Data availability*

The mass spectrometry proteomics data have been deposited to the ProteomeXchange Consortium via the PRIDE[14] partner repository with the dataset identifiers PXD057298 (for rat and human brain homogenate samples) and PXD066265 for LMD samples.

**Fig. S1.** Log2 intensity values of cell-type-specific genes across the different groups (WKY and SHR, (A); and CTR, AD and CAA, (B)). Data distribution is visualized using violin plots with mean intensity (black line). The cell-type-specific gene databases were downloaded from [www.proteinatlas.org](http://www.proteinatlas.org). WKY = Wistar Kyoto, SHR = spontaneously hypertensive rat, CTR = non-demented control, AD = Alzheimer's disease, CAA = cerebral amyloid angiopathy.

Fig. S2. Cd81 and Flot1 are increased in the SHR rats. A) Western blot analysis of Cd81 and Flotillin-1 (Flot1) expression in prefrontal cortex brain homogenates from Wistar Kyoto (WKY) control and spontaneously hypertensive rats (SHR) (n=4 biological replicates). B) and C) Cd81 and Flot1 expression normalized to α-actin. Student t test, *p value < 0.05, **p value < 0.01. Standard error of the mean is included on all column graphs. Soluble brain homogenate sample from non-demented human (Braak 0) was used to verify antibody specificity across species.

**Fig. S3.** Fluorescent intensity does not differ between Hoechst, CD31 and SMα in immunostaining of brain sections from non-demented control and Alzheimer’s disease patients (cohort 2; Fig. 6 in manuscript). A-C) Data from CD81 co-staining experiments in non-demented control (Ctr; blue) and Alzheimer’s disease (AD; orange). N = 4 biological replicates in each group. Student t test, ns = non-significant. Standard error of the mean is included on all column graphs.

**Fig. S4.** Alternative representation of artery-specific CD81 localization. Fluorescent intensity images of Hoechst (blue), CD31 (purple), CD81 (Rainbow LUT or inverse LUT) and SMα (green) from Wistar Kyoto rat (WKY, A), spontaneously hypertensive rat (SHR, B), non-demented control (CTR, C), Alzheimer’s disease (AD, D). The artery wall is highlighted by dotted line. Scale bar = 20 µm.

Supporting Table S1. Demographics of cohort 2.

| Supplementary Table 2: Demographics of cohort 2. | | | | | | | |
| --- | --- | --- | --- | --- | --- | --- | --- |
| Group | Region | Braak stage | Age | Sex | PMI | APOE | APOE4 exp |
| *Control 1* | *BA46* | 0 | 80 | M | 06:30:00 | 3 | 0 |
| *Control 1* | *BA46* | 0 | 83 | F | 08:00:00 | 3 | 0 |
| *Control 1* | *BA46* | 0 | 73 | M | 01:00:00 | 3 | 0 |
| *Control 1* | *BA46* | 0 | 64 | F | 08:35:00 | 4 | 1 |
| *Control 1* | *BA46* | 0 | 70 | M | 07:45:00 | 4 | 1 |
| *Control 1* | *BA46* | 0 | 74 | M | 07:45:00 | 4 | 1 |
| *Control 1* | *BA46* | 0 | 83 | M | 07:50:00 | 3 | 0 |
| *Control 1* | *BA46* | 0 | 78 | M | 04:40:00 | 3 | 0 |
| *Control 1* | *BA10* | 0 | 69 | M | 05:55:00 | 3 | 0 |
| *Control 1* | *BA46* | 0 | 74 | M | 08:00:00 | 3 | 0 |
| *Control 2* | *BA46* | 1 | 83 | M | 04:35:00 | 3 | 0 |
| *Control 2* | *BA10* | 1 | 78 | M | 06:55:00 | 3 | 0 |
| *Control 2* | *BA46* | 1 | 84 | M | 05:35:00 | 3 | 0 |
| *Control 2* | *BA10* | 1 | 79 | M | 05:20:00 | 3 | 0 |
| *Control 2* | *BA46* | 1 | 77 | M | 08:25:00 | 3 | 0 |
| *Control 2* | *BA46* | 1 | 82 | F | 11:30:00 | 3 | 0 |
| *Control 2* | *BA46* | 1 | 82 | F | 05:30:00 | 3 | 0 |
| *Control 2* | *BA46* | 1 | 70 | F | 10:30:00 | 3 | 0 |
| *Control 2* | *BA46* | 1 | 82 | F | 07:00:00 | 3 | 0 |
| *Control 2* | *BA46* | 1 | 91 | F | 07:45:00 | 3 | 0 |
| *Control 3* | *BA46* | 2 | 86 | F | 06:25:00 | 4 | 1 |
| *Control 3* | *BA46* | 2 | 88 | F | 05:40:00 | 3 | 0 |
| *Control 3* | *BA10* | 2 | 85 | M | 05:13:00 | 3 | 0 |
| *Control 3* | *BA46* | 2 | 81 | M | 05:30:00 | 3 | 0 |
| *Control 3* | *BA10* | 2 | 77 | F | 09:15:00 | 4 | 1 |
| *Control 3* | *BA46* | 2 | 82 | M | 05:20:00 | 3 | 0 |
| *Control 3* | *BA46* | 2 | 78 | M | 08:25:00 | 3 | 0 |
| *Control 3* | *BA46* | 2 | 85 | F | 04:40:00 | 4 | 1 |
| *Control 3* | *BA10* | 2 | 89 | F | 05:10:00 | 3 | 0 |
| *Control 3* | *BA46* | 2 | 85 | M | 04:35:00 | 3 | 0 |
| *Control 4* | *BA46* | 3 | 93 | F | 05:35:00 | 3 | 0 |
| *Control 4* | *BA46* | 3 | 87 | F | 07:20:00 | 3 | 0 |
| *Control 4* | *BA46* | 3 | 88 | M | 11:10:00 | 4 | 1 |
| *Control 4* | *BA46* | 3 | 90 | F | 04:45:00 | 3 | 0 |
| *Control 4* | *BA46* | 3 | 81 | M | 06:00:00 | 4 | 1 |
| *Control 4* | *BA46* | 3 | 74 | M | 05:00:00 | 4 | 1 |
| *Control 4* | *BA46* | 3 | 87 | M | 06:05:00 | 3 | 0 |
| *AD1* | *BA46* | 4 | 58 | F | 05:15:00 | 3 | 0 |
| *AD1* | *BA46* | 4 | 86 | F | 05:55:00 | 4 | 1 |
| *AD1* | *BA46* | 4 | 84 | F | 04:50:00 | 4 | 1 |
| *AD1* | *BA10* | 4 | 86 | F | 04:10:00 | 3 | 0 |
| *AD1* | *BA10* | 4 | 85 | F | 03:35:00 | 3 | 0 |
| *AD1* | *BA46* | 4 | 79 | F | 05:40:00 | 4 | 1 |
| *AD1* | *BA46* | 4 | 81 | M | 04:50:00 | 4 | 1 |
| *AD1* | *BA46* | 4 | 86 | M | 06:00:00 | 3 | 0 |
| *AD1* | *BA46* | 4 | 93 | M | 05:50:00 | 3 | 0 |
| *AD1* | *BA10* | 4 | 83 | M | 05:15:00 | 3 | 0 |
| *AD2* | *BA46* | 5 | 77 | F | 06:05:00 | 3 | 0 |
| *AD2* | *BA10* | 5 | 92 | F | 03:45:00 | 3 | 0 |
| *AD2* | *BA10* | 5 | 87 | F | 03:50:00 | 3 | 0 |
| *AD2* | *BA46* | 5 | 84 | F | 04:10:00 | 3 | 0 |
| *AD2* | *BA46* | 5 | 80 | F | 04:30:00 | 4 | 1 |
| *AD2* | *BA46* | 5 | 82 | M | 04:15:00 | 4 | 1 |
| *AD2* | *BA10* | 5 | 88 | M | 05:10:00 | 4 | 1 |
| *AD2* | *BA46* | 5 | 78 | M | 07:45:00 | 4 | 1 |
| *AD2* | *BA46* | 5 | 82 | M | 05:05:00 | 3 | 0 |
| *AD2* | *BA46* | 5 | 63 | M | 06:00:00 | 4 | 1 |
| *AD3* | *BA10* | 6 | 65 | F | 05:40:00 | 3 | 0 |
| *AD3* | *BA46* | 6 | 78 | F | 05:30:00 | 4 | 1 |
| *AD3* | *BA46* | 6 | 81 | F | 06:00:00 | 3 | 0 |
| *AD3* | *BA46* | 6 | 82 | F | 03:15:00 | 4 | 1 |
| *AD3* | *BA10* | 6 | 80 | F | 05:20:00 | 4 | 1 |
| *AD3* | *BA46* | 6 | 82 | M | 03:25:00 | 4 | 1 |
| *AD3* | *BA46* | 6 | 70 | M | 04:23:00 | 4 | 1 |
| *AD3* | *BA10* | 6 | 78 | M | 09:55:00 | 3 | 0 |
| *AD3* | *BA46* | 6 | 83 | M | 03:15:00 | 3 | 0 |
| *AD3* | *BA46* | 6 | 86 | M | 03:25:00 | 4 | 1 |
| *AD-CAA* | *BA10* | 5 | 67 | M | 05:22:00 | 4 | 1 |
| *AD-CAA* | *BA46* | 4 | 89 | F | 03:05:00 | 3 | 0 |
| *AD-CAA* | *BA10* | 5 | 88 | F | 05:05:00 | 4 | 1 |
| *AD-CAA* | *BA46* | 5 | 82 | F | 04:25:00 | 3 | 0 |
| *AD-CAA* | *BA46* | 5 | 75 | F | 06:00:00 | 4 | 1 |

Supporting Table S3. Description of 24 candidate proteins. Description and presence of candidate proteins in NeuroPro Alzheimer’s disease (AD) database[11].

| Candidate proteins | NeuroPro Score (Total) | NeuroPro Score (Bulk Tissue) | Increased in AD | Decreased in AD | Single Directional Outlier | Inconsistent in AD | NFT Protein | Plaque Protein | Inconsistent in Plaques | CAA Protein | Published Studies Linked to AD | Understudied |
| --- | --- | --- | --- | --- | --- | --- | --- | --- | --- | --- | --- | --- |
| ACAT1 | 3 | NA |  |  |  |  | PRESENT | PRESENT |  |  |  |  |
| NME1 | 4 | 3 |  |  |  |  |  | PRESENT |  |  |  |  |
| METTL7A | 4 | 2 |  |  |  |  |  | PRESENT |  |  |  |  |
| MAPT | 16 | 11 | TRUE |  |  |  | INCREASED | INCREASED |  |  | 23171 |  |
| GNG7 | 5 | 3 |  |  |  |  |  | PRESENT |  |  |  |  |
| FLOT2 | 4 | 2 |  |  |  |  |  | PRESENT |  |  |  |  |
| FLOT1 | 3 | NA |  |  |  |  | PRESENT | PRESENT |  |  |  |  |
| PIP4K2C | 3 | 1 |  |  |  |  |  | PRESENT |  |  |  |  |
| TPM3 | 7 | 4 |  |  |  |  | PRESENT | PRESENT |  |  |  |  |
| TOM1L2 | 8 | 6 |  | TRUE |  |  |  | PRESENT |  |  |  |  |
| SPTAN1 | 7 | 4 |  |  |  |  | PRESENT | PRESENT |  |  |  |  |
| SEPTIN7 | 8 | 5 |  | TRUE |  |  | PRESENT | PRESENT |  |  |  |  |
| RDH13 | 5 | 5 |  | TRUE |  |  |  |  |  |  |  |  |
| ESD | 9 | 7 | TRUE |  |  |  |  | PRESENT |  |  |  |  |
| ATIC | 5 | 3 |  |  |  |  |  | PRESENT |  |  |  |  |
| ANXA11 | 3 | 1 |  |  |  |  |  | PRESENT |  |  |  |  |
| ATOX1 | 1 | 1 |  |  |  |  |  |  |  |  |  |  |
| ADGRG1 | 4 | 4 |  |  |  |  |  |  |  |  |  |  |
| ADAM22 | 6 | 4 |  |  |  |  |  | PRESENT |  |  |  |  |
| ATP2B3 | 7 | 5 |  | TRUE |  |  |  | PRESENT |  |  |  |  |
| COX6A1 | NA | NA |  |  |  |  |  |  |  |  |  |  |
| CD81 | 4 | 3 |  |  |  |  |  | PRESENT |  |  |  |  |
| BDH1 | 6 | 4 |  |  |  |  |  | PRESENT |  |  |  |  |
| B2M | 3 | 3 |  |  |  |  |  |  |  |  |  |  |

**Supporting materials references**

[1]	Bastrup JA, Rippe C, Swärd K, Jepps T (2026) Temporal Proteomic Remodeling of Cerebral Arteries in Male Hypertensive Rats. *Hypertension*.

[2]	van der Horst J, Manville RW, Hayes K, Thomsen MB, Abbott GW, Jepps TA (2020) Acetaminophen (Paracetamol) Metabolites Induce Vasodilation and Hypotension by Activating Kv7 Potassium Channels Directly and Indirectly. *Arterioscler Thromb Vasc Biol* **40**, 1207–1219.

[3]	Montine TJ, Phelps CH, Beach TG, Bigio EH, Cairns NJ, Dickson DW, Duyckaerts C, Frosch MP, Masliah E, Mirra SS, Nelson PT, Schneider JA, Thal DR, Trojanowski JQ, Vinters H V., Hyman BT (2012) National Institute on Aging–Alzheimer’s Association guidelines for the neuropathologic assessment of Alzheimer’s disease: a practical approach. *Acta Neuropathol* **123**, 1–11.

[4]	Bastrup JA, Aalkjær C, Jepps TA (2022) Identification of novel proteins and mechanistic pathways associated with early-onset hypertension by deep proteomic mapping of resistance arteries. *J Biol Chem* **298**, 101512.

[5]	Wiśniewski JR, Zougman A, Nagaraj N, Mann M (2009) Universal sample preparation method for proteome analysis. *Nat Methods* **6**, 359–362.

[6]	Bastrup JA, Jepps TA (2023) Proteomic mapping reveals dysregulated angiogenesis in the cerebral arteries of rats with early-onset hypertension. *J Biol Chem* 105221.

[7]	Demichev V, Messner CB, Vernardis SI, Lilley KS, Ralser M (2020) DIA-NN: neural networks and interference correction enable deep proteome coverage in high throughput. *Nat Methods* **17**, 41–44.

[8]	Tyanova S, Temu T, Sinitcyn P, Carlson A, Hein MY, Geiger T, Mann M, Cox J (2016) The Perseus computational platform for comprehensive analysis of (prote)omics data. *Nat Methods* **13**, 731–740.

[9]	Szklarczyk D, Franceschini A, Wyder S, Forslund K, Heller D, Huerta-Cepas J, Simonovic M, Roth A, Santos A, Tsafou KP, Kuhn M, Bork P, Jensen LJ, von Mering C (2015) STRING v10: protein–protein interaction networks, integrated over the tree of life. *Nucleic Acids Res* **43**, D447–D452.

[10]	Shannon P, Markiel A, Ozier O, Baliga NS, Wang JT, Ramage D, Amin N, Schwikowski B, Trey I (2003) Cytoscape: A Software Environment for Integrated Models of Biomolecular Interaction Networks. *Genome Res* **13**, 2498–2504.

[11]	Askenazi M, Kavanagh T, Pires G, Ueberheide B, Wisniewski T, Drummond E (2023) Compilation of reported protein changes in the brain in Alzheimer’s disease. *Nat Commun* **14**, 4466.

[12]	Lee S, Jang K, Lee H, Jo YS, Kwon D, Park G, Bae S, Kwon YW, Jang J, Oh Y, Lee C, Yoon JH (2024) Multi‐proteomic analyses of 5xFAD mice reveal new molecular signatures of early‐stage Alzheimer’s disease. *Aging Cell* **23**, 1–17.

[13]	Koopmans F, van Nierop P, Andres-Alonso M, Byrnes A, Cijsouw T, Coba MP, Cornelisse LN, Farrell RJ, Goldschmidt HL, Howrigan DP, Hussain NK, Imig C, de Jong APH, Jung H, Kohansalnodehi M, Kramarz B, Lipstein N, Lovering RC, MacGillavry H, Mariano V, Mi H, Ninov M, Osumi-Sutherland D, Pielot R, Smalla K-H, Tang H, Tashman K, Toonen RFG, Verpelli C, Reig-Viader R, Watanabe K, van Weering J, Achsel T, Ashrafi G, Asi N, Brown TC, De Camilli P, Feuermann M, Foulger RE, Gaudet P, Joglekar A, Kanellopoulos A, Malenka R, Nicoll RA, Pulido C, de Juan-Sanz J, Sheng M, Südhof TC, Tilgner HU, Bagni C, Bayés À, Biederer T, Brose N, Chua JJE, Dieterich DC, Gundelfinger ED, Hoogenraad C, Huganir RL, Jahn R, Kaeser PS, Kim E, Kreutz MR, McPherson PS, Neale BM, O’Connor V, Posthuma D, Ryan TA, Sala C, Feng G, Hyman SE, Thomas PD, Smit AB, Verhage M (2019) SynGO: An Evidence-Based, Expert-Curated Knowledge Base for the Synapse. *Neuron* **103**, 217-234.e4.

[14]	Perez-Riverol Y, Csordas A, Bai J, Bernal-Llinares M, Hewapathirana S, Kundu DJ, Inuganti A, Griss J, Mayer G, Eisenacher M, Pérez E, Uszkoreit J, Pfeuffer J, Sachsenberg T, Yılmaz Ş, Tiwary S, Cox J, Audain E, Walzer M, Jarnuczak AF, Ternent T, Brazma A, Vizcaíno JA (2019) The PRIDE database and related tools and resources in 2019: improving support for quantification data. *Nucleic Acids Res* **47**, D442–D450.
